# Cost-benefit analysis of the offshore wind power transition in the Mediterranean: Towards a “New Eldorado”?

**DOI:** 10.12688/openreseurope.19339.1

**Published:** 2025-03-07

**Authors:** Marta Avesani

**Affiliations:** 1 EUROSUD Programme, University of Glasgow School of Social and Political Sciences, Glasgow, Scotland, G12 8RT, UK

**Keywords:** Mediterranean Sea, Green Deal, Barcelona Convention, Offshore Wind Energy, Floating Technology

## Abstract

The condition of the environment remains at the forefront of contemporary debates among political leaders and societal stakeholders, particularly in the context of addressing climate change. Offshore Wind Farms (OWFs) are emerging as a promising pathway for renewable energy development in the Mediterranean. While existing literature extensively explores the potential of OWF technology in advancing climate neutrality targets by 2030, comparatively little attention has been given to the challenges and trade-offs associated with its implementation. This essay examines the costs and benefits of deploying OWFs in the Mediterranean, assessing their economic, environmental, and social impacts through a trade-off analysis. Despite regulatory and environmental challenges, the deployment of OWFs in the Mediterranean is set to expand, driven by low marginal costs, favourable climatic and geographic conditions, and growing interest from international investors. The study underscores the urgent need for comprehensive legislation to address environmental, social, and economic risks, critically evaluating gaps in the current legal framework and examining the opportunities and challenges associated with renewable energy expansion. The analysis serves as a valuable resource for policymakers, investors, and environmental advocates, providing a framework to balance environmental preservation with the expansion of renewable energy. Addressing these critical considerations aims to support the creation of strategies that foster a harmonious integration of renewable energy projects within the Mediterranean region’s environmental and socio-economic landscape.

## Introduction

As the global energy transition becomes increasingly critical, debates and studies on renewable energies are gaining renewed attention. Seas and oceans act as vast energy collectors, primarily through solar energy, which can be converted into potential, kinetic, and thermal energy. The Mediterranean Sea, capturing substantial solar energy and generating water currents, offers opportunities for energy production via submerged turbines, transforming renewable and inexhaustible energy into electricity. Wind energy dominates the Mediterranean's renewable potential, especially with floating wind turbine technology advancements. However, despite existing measures in place, Europe’s natural environment is degrading rapidly, and it necessitates constant legislative updates to align urban and industrial growth with environmental sustainability ^1^. In this respect, sustainable development focuses on fulfilling current needs while ensuring future generations can satisfy theirs ^2^. It requires economic efficiency, social equity, and environmental sustainability while integrating ecological concerns with societal and financial priorities ^3^. Renewable energy derives from inexhaustible and continuously replenished sources, and it offers a crucial response to the climate crisis. In the Mediterranean, offshore wind energy offers a promising solution.

The European Union (EU) green transition has been shaped by various legislations and agreements, such as the EU’s Renewable Energy Directive (RED) ^4^, followed by Directive (EU) 2018/2001, which promotes the use of energy from renewable sources, ensuring alignment with recent sustainability goals ^5^. Renewable energy is vital for reducing greenhouse gas emissions in line with COP26 and COP27, focusing on advancing the Paris Agreement's goals ^6^. The RED and its following updates provide a legal framework to remove barriers to a clean energy transition, encouraging renewable heating, cooling, and transport fuels while promoting investments and lowering renewable energy technology costs ^7^. In 2011, the European Commission (EC) introduced the Energy Roadmap 2050 to cut EU greenhouse gas emissions by 80–95% in 2050 compared to 1990 ^8^. This was followed by the 2030 Energy and Climate Framework in 2014, which set targets to reduce emissions by at least 40%, increase renewable energy use, and achieve a 27% improvement in energy efficiency by 2030 ^9^. The European Green Deal, announced in 2019, introduced the EU Adaptation Strategy, replacing the Energy Roadmap 2050. To meet climate goals, a 90% reduction in transport-related greenhouse gas emissions is needed, alongside a targeted increase in inland waterways and short-sea shipping by 25% by 2030 and 50% by 2050 ^10^. However, this will significantly affect marine and coastal environments.

Offshore wind energy, in the form of offshore wind farms, is expected to become a key trend in wind energy production in the Mediterranean. Wind energy remains the EU's second-largest source of power generation capacity, coming close to matching gas installations ^11^. Offshore wind turbines, either seabed-fixed or floating, gain advantages from stronger, more consistent, and more frequent winds than land-based sources turbines ^12^. As a result, EU legislation has sought to address this emerging sector. In 2020, the EC published a strategy for developing marine renewable energy. In the same year, installed offshore wind capacity in the EU-27 was estimated at 12 GW, and according to forecasts, the EC aims to reach 300 GW of offshore wind capacity in 2050, with an intermediate step of 60 GW in 2030 ^13^. This epochal shift is anticipated to occur at an unprecedented rate for renewables since, under current policies, the projected installation capacity for 2050 would only be about 90 GW ^14^. Achieving such targets necessitates the collaboration of all Member States by developing national low-carbon roadmaps. Significant cuts in carbon emissions will also save fossil fuel imports, enhance air quality, and reduce climate change risks as part of this ambitious global action ^15^.

Growing international interest, reduced costs, and favourable conditions signal a transformative opportunity. Although no operational OWFs exist in the Mediterranean ^16^, the French Government has taken the lead in the Mediterranean by approving the installation of a pilot floating offshore wind farm, known as “Eoliennes Flottantes du Golfe du Lion” (EFGL) and two additional pilot projects, while Spain is following suit with the development of the Tramuntana Floating Offshore Wind Farm Project ^17^. These projects are part of national plans outlined by the European Directive 2014/89/EU ^18^. The Mediterranean's deep waters and strong winds are well-suited to this innovation, which can access 80% of the world’s wind resources in waters deeper than 50 meters ^19^. The first OWFs were installed in Northern Europe and essentially transposed onshore wind farms to the sea, where the turbines are fixed on masts implanted in the sea bed ^20^. However, the traditional technique has several limitations, notably related to the depth of the implantation zone, which must not exceed 50 metres (m). As a result, they are usually located near the coast, where several environmental risks are identified, such as visual impacts on the landscape and those associated with coastal fishing and commercial shipping ^21^. 

Given the unique characteristics of the Mediterranean Sea, cost-benefit analyses remain speculative due to the lack of concrete operational examples. Research focuses on OWFs in the North, Baltic, and North Atlantic, which differ from the Mediterranean's diverse shelf morphology and fragile benthic habitats like seagrass meadows and coralligenous reefs ^22^. The limited understanding of OWFs affects the Mediterranean environment, promoting an excessively hopeful perception of OWFs as vital contributors to the green transition while neglecting substantial risks. The long-term impacts of OWFs on marine safety remain insufficiently assessed, with concerns ranging from pollutants released by turbines, buoys, and mooring systems to adverse effects on marine ecosystems and socio-economic conditions. These risks could ultimately undermine the broader objectives of the energy transition. Through a literature review, this essay firstly examines the legislative framework surrounding OWF technology, highlighting its gaps. Analysing the environmental, social, and economic challenges posed by OWFs, it offers a comprehensive understanding of the obstacles to their implementation. The findings stress that inadequate legislation could restrict the benefits of OWFs, leaving social and economic damages unaddressed. The essay illustrates how the energy transition faces significant challenges, revealing that even renewable energy production is not as environmentally benign as often assumed.

## EU legislation on sustainable use of marine resources

Since the Rio Conference in 2012, the international community has been actively addressing the sustainable use of marine resources. Building on the foundation of the 1992 Rio Declaration on Environment and Development, Sustainable Development Goal (SDG) 14 was adopted in 2015, aiming to “conserve and sustainably use the oceans, seas, and marine resources for sustainable development” ^23^. The Mediterranean Action Plan (MAP) was established in 1975 as a multilateral environmental agreement under the United Nations Environment Programme (UNEP). Its primary goal is to protect the marine environment through a coordinated regional approach ^24^. Over time, UNEP/MAP has evolved into a global governance model, which is key in facilitating the negotiation and adoption of the Convention for the Protection of the Marine Environment and the Coastal Region of the Mediterranean (Barcelona Convention) and its associated protocols. With seven protocols under the MAP, the Barcelona Convention is the region’s key legally binding multilateral environmental agreement ^25^, which came into force in 2004. Its Contracting Parties (CP) consist of 21 Mediterranean countries and the EU, encompassing the Mediterranean Sea eastward from the Strait of Gibraltar. The Convention allows each party to extend its application to its national territory, ensuring a broad and adaptable scope for regional cooperation.

Cooperation mechanisms, such as those under the Barcelona Convention, aim to coordinate the use of maritime resources, strengthen governance, and foster economic growth while promoting sustainable practices ^26^. These efforts align with broader frameworks of renewable energy policies and marine ecosystem protections. According to Art.4, the CPs agree to take all necessary measures, individually or jointly, to prevent, reduce, and eliminate pollution of the Mediterranean Sea and to protect the marine environment to support sustainable development ^27^. A key component is the Dumping Protocol, entered into force in 1978, and amended in 1995 ^28^. Excluding Montenegro, all CPs to the Barcelona Convention are also parties to this protocol, which prohibits dumping activities, incineration at sea, and waste disposal, except for specific materials ^29^. In 2021, during the 22nd Meeting of the CPs in Turkey, a decision was made to amend the Annex to the Dumping Protocol. These amendments, binding in the EU under Art. 29 of the Barcelona Convention, revised the criteria for issuing permits for disposal at sea. The updated criteria outline the characteristics and composition of dumped materials and the attributes of dumping sites and deposit methods ^30^. This reflects the CPs' commitment to reducing pollution and ensuring sustainable management of the Mediterranean Sea.

In the Mediterranean, efforts are guided by the Mediterranean Strategy for Sustainable Development (MSSD), adopted during COP19 of the Barcelona Convention in 2016. The MSSD provides a framework to integrate regional, sub-regional, and national policies with the 2030 Agenda for Sustainable Development ^31^. This approach aligns with the EU Marine Strategy Framework Directive (MSFD) 2016–2025, which states that ecosystem protection is a cornerstone of economic and environmental policy ^32^. It builds on this by targeting the Good Environmental Status (GES) of marine and coastal environments. The MSFD fosters ecosystem-based management, harmonising efforts among 23 coastal and five landlocked EU states and coordinating with non-EU countries to sustainably manage a 5.72 million km² marine area ^33^. In 2018, the CPs of the Barcelona Convention adopted the Ecosystem Approach (EcAp) and agreed on a roadmap for its implementation in the Mediterranean. The EcAp is a strategy for the integrated management of land, water, and living resources that promotes their conservation and equitable use. As such, it is the guiding principle for policy development and implementation undertaken under the auspices of the Barcelona Convention, such as achieving GES in the Mediterranean marine and coastal environment through informed management decisions based on integrated quantitative assessment and monitoring ^34^. At the Mediterranean level, the EcAp and the MSFD are implemented in synergy and coherence, and they are among the most ambitious international frameworks for marine environmental protection ^35^. Additional measures include Art.9 of the Integrated Coastal Zone Management Protocol (ICZM) and the Union for the Mediterranean's 2015 Ministerial Declaration, all of which emphasise ecosystem preservation. EU policies, such as the Water Framework Directive (WFD), further reinforce these efforts by aiming to achieve good ecological status for water bodies. While its initial goals were delayed due to funding and implementation challenges, the WFD established a governance framework for integrated water management, reducing chemical pollution and linking human activities to their environmental impacts ^36^.

This integrated approach ensures marine ecosystems remain functional and resilient for future generations. After launching the world’s first global framework to finance a sustainable Ocean Economy ^37^, the EU introduces its new strategy on Biodiversity for 2030, focusing on “making nature healthy again” ^38^. The strategy boosts the protection of marine ecosystems by expanding the protected areas and stressing the importance of the EcAp for managing human activities at sea. Sustainable innovation presents significant opportunities in green ports, shipping, aquaculture, water management, conservation, recycling, and maritime ecotourism, all key to the region's future. Meanwhile, the Commission emphasised the benefits of sustainable blue economy activities, advocating for an international Blue Economy Sustainability Framework ^39^. However, according to the results of the 2020 report on the first implementation cycles of the MSFD, the EU framework for protecting the marine environment needs to be strengthened ^40^. Indeed, GES is achievable only if the pressures on the marine and coastal environment decrease and, therefore, if the uses of the marine and coastal environment that cause these pressures are adjusted. Surprisingly, the notion of GES appears only once in the text of the European Green Deal and only once in the Commission’s Communication on the Sustainable Blue Economy ^41^. This may indicate a persistent lack of integration of marine protection into broader policies or a dominance of economic priorities over marine conservation, even under the guise of climate change adaptation.

 To address this, the EU approved a taxonomy defining criteria for economic activities contributing to climate adaptation and prioritising adaptation solutions ^42^. Furthermore, aware of the scale and diversity of its natural heritage, the EU has been very attentive to preserving its ecosystems and has created an extensive body of legislation to protect and enhance Mediterranean biodiversity. The Nature Directives were made to ensure a healthy natural world in the EU through a legal framework for protecting species and natural habitats. These directives establish the Natura 2000, a network of nature protection areas comprising Special Areas of Conservation and Special Protection Areas designated under the Habitats Directive ^43^ and the Birds Directive ^44^. Natura 2000 ensures the conservation of rare, threatened, or endemic species, including all wild bird species in the EU, through protection, management, and monitoring measures. It covers 18% of the EU's land area and 6% of its sea area and is currently predominantly active on land ^45^. Still, significant gaps are present concerning the marine environment, and only 50% of all Natura sites have management plans with conservation objectives and measures. In 2014, the European Commission conducted a comprehensive evaluation of the Nature Directives, known as the “Fitness Check,” to assess their effectiveness ^46^. The evaluation confirmed that the directives are fit for purpose and capable of improving species and habitat conditions; however, their success depends on practical implementation.

### EU legislation on OWFs

When discussing wind energy, a key distinction is between energy generated by onshore and offshore wind farms. OWFs consist of multiple wind turbines that convert the wind's kinetic energy into electricity ^47^. In the Mediterranean, OWFs are vital to the Blue Economy, a sector generating significant regional income. The Blue Economy encompasses traditional maritime activities such as fishing, aquaculture, coastal tourism and emerging sectors such as renewable energy and marine biotechnologies ^48^. The Sustainable Blue Economy focuses on clean technologies, renewable energy, and circular material flows to secure long-term social and economic stability within planetary boundaries ^49^. It seeks to balance the optimal use of maritime natural capital with human and social capital, advancing sustainable development, social equity, and reduced ecological risks. The EU's taxonomy for climate change mitigation, outlined in Regulation (EU) 2020/852 and amending Regulation (EU) 2019/2088, provides a framework for determining whether economic activities and investments qualify as environmentally sustainable, including provisions for the marine environment ^50^. The regulation focuses on transitioning to renewable energy, reducing fossil fuel reliance, and promoting carbon-neutral energy sources. It allows activities with no viable low-carbon alternatives as long as they limit global temperature rise to 1.5°C above pre-industrial levels. Such activities must comply with strict GHG emissions standards and support the development of low-carbon alternatives.

Commission Delegated Regulation (EU) 2021/2139 establishes the general framework for determining whether an economic activity and its investments qualify as environmentally sustainable and includes several chapters related to the marine environment ^51^. It ensures the conservation of rare, threatened, and endemic species, including all wild bird species in the EU, through measures for their protection, management, and monitoring. It also highlights the restoration of wetlands and the development of renewable energy sources such as wind, ocean, hydropower, geothermal energy, and cogeneration technologies. Furthermore, it states that offshore wind projects must not hinder achieving GES as defined by the MSFD, requiring measures to prevent or mitigate impacts on biodiversity and seabed integrity, supported by an environmental impact assessment and necessary mitigation and compensation measures. However, the references to the proper use of offshore wind turbines contained in the Regulation are insufficient to address their wide-ranging environmental impacts, including harm to marine ecosystems and pollutant emissions during their creation and dismantling. Furthermore, their buoys or mooring systems could contain one or more environmentally hazardous substances.

Natura 2000 sites and the Special Protection Areas for birds, considered the wealthiest areas in terms of biodiversity, must be avoided and preserved from the implantation of OWFs, notably because if their integrity were affected, their legal status would be at risk. These areas, rich in flora and fauna of immense heritage value, are subject to a strict obligation to ensure their protection, with the risk of condemnation by the member states involved in case of failure ^52^. The Habitats Directive does not prohibit wind farms in Natura 2000 sites but requires case-by-case analysis for their siting. An assessment must demonstrate that the project will not have significant adverse impacts on the site, particularly on habitats, including direct loss, degradation, fragmentation, species disturbance, or other indirect effects on environmental quality ^53^. The study must consider the extent and magnitude of the impacts and assess different active projects in the area. Indeed, if well combined with other economic activities, such as those linked to other renewable energy sources, the negative impacts of offshore wind energy installations could be reduced. At the same time, biodiversity in these zones may even increase. However, there is no legal basis at the national or EU level to prevent such an occurrence.

The EC guidance document on wind energy developments and Natura 2000 sites was designed to align with new EU policies and legislation on renewable energy and offshore wind technology ^54^. It clarifies the application of the Natura Directives, updates the 2011 guidance on wind farms in Natura 2000 sites, and highlights the need for further guidance, knowledge, and best practices to reconcile the objectives of different policies, underscoring the importance of further exploration in this area. However, although official documents, EC communications are non-legally binding ^55^. Thus, the guidance document is not legally enforceable; its primary aim is to inform about particular elements of relevant EU legislation, especially regarding the effects of wind energy on biodiversity and effective practices to mitigate these effects ^56^. Its non-binding nature risks limiting credibility and enforcement. Therefore, it is necessary to carry out a case-by-case analysis before building these constructions. This highlights gaps in EU legislation, where progress in one area may hinder others.

## Environmental challenges

As the offshore wind industry grows, its impacts on marine ecosystems remain insufficiently explored. The estimated 25-to-30-year lifecycle of an OWF encompasses multiple stages, including raw material extraction, component manufacturing, installation, maintenance, and decommissioning ^57^. OWFs reduce emissions but can harm aquatic ecosystems, with biodiversity protection often overlooked in offshore energy areas. Floating technology holds significant potential as it overcomes the limitations of traditional wind farms by harnessing stronger, more consistent winds ^58^. Floating wind turbines, typically with horizontal axes and two or three blades, are anchored to the seabed by cables and towed into position. These turbines can have capacities of 15 MW or more, with blade lengths exceeding 100 m, and are installed in clusters connected by seabed cables ^59^. They can operate in any water depth, allowing them to be placed across the continental shelf, far from shore, avoiding visual and logistical issues linked to coastal fishing and shipping. The environmental impact of electricity generation from a floating wind power plant is not significantly different from that of conventional offshore wind power plants, provided that it operates with a higher capacity factor ^60^. However, floating technology introduces additional risks, including those from suction anchors, subsea cables, and ballast water in internal tanks ^61^. It may have a larger environmental footprint due to potential repairs onshore ^62^. Underwater cables could limit accessible fishing zones ^63^, and debates persist over the potential loss of biodiversity in benthic and pelagic habitats, which house diverse marine life, from molluscs and sponges to fish and birds ^64^. To mitigate these impacts, policymakers must apply the Precautionary Principle ^65^, as outlined in the Treaty on the Functioning of the European Union and reflected in the MSFD and the EU Maritime Spatial Planning Directive (MSPD), which promotes sustainable maritime growth ^66^.

Impact assessments should address all OWF development stages. Key concerns include visual and acoustic stress, habitat loss, pollution, water turbidity, bird collisions, electric fields, artificial reefs, and maintenance effects ^67^. Additionally, marine traffic and OWF maintenance threaten marine fauna like mammals and sea turtles, with the northern Mediterranean and the Strait of Gibraltar being key hotspots for ship strikes ^68^. Marine fauna, particularly mammals, rely on sound for essential survival activities such as communication, prey identification, and orientation. Human-made noise from shipping, construction, and military operations disrupts vital functions. It harms animal welfare, with pile driving for offshore wind farms being a significant source of undersea noise pollution ^69^, causing physical stress and injuries and alter marine population dynamics. Macroscopic pollution from household and industrial waste and chemical pollution from toxic substances like oil spills and underwater corrosion harm aquatic life. During OWF construction and operation, waste discharge also poses risks, including entanglement ^70^. Toxic metals from sacrificial anodes that protect submerged structures can dissolve into the environment, forming toxic bonds with other elements ^71^. Risks include accidents at turbine sites caused by storms, fires, or ship collisions, which could cause oil or chemical spills. Wind turbine accidents are thus a significant concern ^72^.

Electromagnetic fields generated by underwater power cables transmitting electricity from OWFs to the coast have serious impacts on marine ecosystems ^73^. The primary concern is the effect on animals that use the Earth's geomagnetic field for orientation and rely on electroreception to hunt, with potential disruptions to these critical functions ^74^. Furthermore, energy transmission through cables generates heat, raising their temperature to 70°C and warming the surrounding environment ^75^. When cables are laid above the seabed, convection transfers heat to the surrounding seawater, impacting the seabed ecosystem depending on the cable type and proximity. Additionally, artificial lights used during construction, operation, maintenance, or at night for obstruction warnings can pose a threat, especially to nocturnal migratory birds and sea turtles. These species may become disoriented and attracted to the turbines, leading to potential collisions between seabirds or migratory birds and wind turbines, especially when birds pass through areas swept by rotors ^76^. Actual fatalities are hard to track, so researchers focus on collision and avoidance rates to estimate the risk. Sensitivity maps can help identify areas with high collision potential, aiding strategic planning, while temporary turbine shutdowns during mass migration events have effectively reduced risks ^77^. Identifying at-risk species requires considerable resources, with new systems using radar and cameras for real-time bird detection. Other strategies include altering blade patterns, using ultraviolet-reflecting paints, and deploying bird deterrents ^78^.

Offshore wind turbines, typically installed at depths of up to 50 meters, are often placed in coastal areas rich in marine biodiversity, leading to significant environmental impacts. Birds are attracted to the bases of turbines, increasing the risk of collision with rotating blades, which can disrupt migration routes ^79^. Many seabirds in the Mediterranean, some endangered or threatened ^80^, breed in the region before migrating to the Atlantic. Additionally, wind farms pose collision risks to migratory and local bats. Turtles and other mammals are also at risk from construction and turbine operations, such as collisions or entanglement with submarine cables. Furthermore, new artificial substrates can introduce non-native species, disrupting local biodiversity ^81^. OWF anchors and cables can disturb Maerl and seagrass habitats, leading to habitat loss and displacement of species. Geotechnical construction methods can also remobilise nutrients and contaminants, further impacting species' survival and Maerl, which provide shelter for marine organisms and act as carbon reservoirs ^82^. Seagrass meadows, especially those formed by the endangered Posidonia oceanica ^83^, are crucial ecosystems that support fish, molluscs, and other species ^84^.

Secondary impacts of OWFs are those not directly caused by the turbines but resulting from their presence. The “reef effect” is generated when the construction of OWFs creates new artificial substrates, particularly turbine foundations, which become artificial reefs ^85^. These reefs attract new marine life, especially in sandy habitats, boosting local biodiversity. While native species colonise these reefs, some species may be absent, and mobile species, like fish and crabs, may need to relocate due to lost habitat ^86^. This effect can benefit fish and crustaceans, though outcomes depend on the type of reef and native species characteristics, as invasive species may spread through these artificial reefs. The EC guidelines stress that impacts in protected areas must not harm habitats' or species' integrity or conservation status ^87^. The “reserve effect” increases fish survival rates due to restricted fishing near OWFs, though higher fishing pressure may negate the “spillover effect” of no-fishing zones ^88^. Moreover, offshore wind turbines can reduce wind speed downstream, increasing turbulence and altering ocean dynamics. They may also cause upwelling and downwelling around the wind farm. Anchoring systems can stir up suspended sediments, leading to persistent water turbidity and bathymetry changes. Pollution from turbines or the dissolution of sacrificial anodes can further degrade water quality. However, long-term effects remain insufficiently studied, and a comprehensive and reliable assessment of these cumulative impacts is needed ^89^.

## Socio-economic challenges

Little research and monitoring exists on the socio-economic impacts of OWFs in the Mediterranean, which are often overshadowed by environmental and economic concerns. Cumulative impacts include effects on employment, economic growth, housing, local services, community welfare, and conflicts with sectors like tourism and fisheries. From an economic perspective, OWFs affect employment, Gross Value Added (GVA), and coastal industries such as fishing, shipping, and recreation. OWF construction and maintenance phases generate jobs, focusing on training programs and local supply chain opportunities ^90^. They improve infrastructure, especially ports, and generate socio-economic benefits during construction. However, social impacts include housing pressures and quality of life, with developers potentially offering housing support. Tourism is a fundamental economic driver in the Mediterranean regions, especially in coastal recreational activities such as sailing, scuba diving, swimming, and bird and whale watching. The creation of OWFs can limit these activities, and the economic revenue losses could be significant ^91^. Many Mediterranean coastal states have narrow continental shelves, and OWFs are planned relatively close to the coast, which can impact the visual image of the seascape to lead tourists to move away from beaches to more natural seascapes, raising concerns for local communities affected by the loss of economic income ^92^. Thus, Community Benefit Agreements recognise local involvement, provided to the community as a gesture of appreciation for their participation in projects deemed to be in the national interest, rather than as direct compensation for local impacts ^93^. Community acceptance of offshore projects is crucial, as residents may perceive these farms as part of industrialising the sea, reinforcing multiple economic worries.

Banning fishing in areas where OWFs are installed could seriously affect this industry, especially the small-scale fisheries in the area, which are widely present in the Mediterranean coastal states ^94^. In addition, fishing is restricted during the farms' construction phases, and the construction settlements could affect fishery resources ^95^. The ban on fishing could also jeopardise the scientific surveys of fishery resources that have been fundamental in evaluating developments in the areas throughout the years, as the farms could increase local underwater biodiversity by excluding these areas from fishing. Sensitive buffer zones between OWFs and MPAs could partially protect sensitive species, although further studies are needed. However, the most efficient turbine installation areas often overlap with multiple MPAs, offering ideal wind and bathymetric conditions. Focusing solely on technical criteria, energy companies may select these sites for OWFs without fully considering potential ecological impacts. Given the high traffic in Mediterranean waters, OWFs pose a risk of collision between commercial and passenger vessels, further reducing navigable space in one of the world's most crowded seas ^96^. Moreover, it augments traffic density on other available routes, increasing the risk of collision.

Wind energy production depends on materials with a high supply risk. The new wind turbines, where a generator converts mechanical energy into electrical energy, are programmed to achieve maximum efficiency and the lowest possible energy costs. Fluctuating wind energy makes variable-speed generators, like permanent magnet (PM) generators, and blades with high strength-to-weight ratios and fatigue resistance the most suitable options. Materials must be resistant to corrosion, wind, and extreme weather conditions. Usually, the main components of the turbines are made of steel and manufactured from a mixture of iron ore, carbon and other elements, including nickel, molybdenum, titanium, manganese, vanadium or cobalt, and the blades are made of fibreglass and resin. However, the production of these components relies heavily on several Rare Earth Elements (REEs), including neodymium-iron-boron and dysprosium, which are primarily sourced outside the EU ^97^, mainly from China (54%) and Latin America (29%) ^98^. Demand for these materials is expected to rise significantly by 2050 compared to current levels ^99^.

Raw materials sourced outside the EU create economic dependence on non-EU countries. This reliance raises concerns about the feasibility and long-term effects of the green transition. While renewable energy costs are decreasing and expected to play a significant role in decarbonisation, the growing demand for minerals will lead to increased extraction, with potential environmental impacts if not managed responsibly, as the mining sector accounts for 2–11% of global energy consumption ^100^. Climate-smart mining practices are necessary to prevent shifting emissions from power generation to mining. Moreover, metal prices, particularly for generators containing REEs, heavily influence wind turbine costs. High international demand and limited availability of raw materials pose risks to the global transition to sustainable energy. The bottleneck goes from a high risk for raw materials to a medium risk for the supply of processed components and is almost undetectable for assemblies. Concerns stem from China's near-monopoly on REEs and PM manufacturing, with potential price fluctuations due to trade disputes, as seen during the 2011 crisis when China imposed strict market restrictions ^101^.

Many European countries have started intense campaigns of seabed exploration to detect the presence of REEs and proceed with their extraction. Polymetallic nodules proliferate in these environments and appear to be the new paradise for REEs ^102^. Extractive activities are costly in terms of machinery used for extraction, mainly because of their impact on marine ecosystems. Under UNESCO’s objective of chart graphing 80% of seabeds by the end of the decade, many exploratory activities have already been performed, which open the way for future extractive activity in the concerned seas ^103^. The UNEP Global Environmental Alert Service reports that massive seabed sulphide deposits containing copper, lead, zinc, silver, and other metals are primarily found under national jurisdiction, with polymetallic nodules in the equatorial Pacific and cobalt-rich crusts in the western Pacific ^104^. Exploring deep seabeds is often followed by exploiting these areas, which are still mysterious and widely unknown. However, deep-sea mining presents environmental threats. Extraction involves pulverising mineral crusts pumped to vessels, releasing pollutants, noise, light, and invasive species. The light affects deep-water species, while the altered water composition reduces oxygen and clouds the environment ^105^. Mining can also destroy vent communities ^106^ which take years to recover ^107^, and suspend sediments, damaging bottom-dwelling species.

To capitalise on valuable discoveries, the rush for control in the Mediterranean has intensified as coastal states establish Exclusive Economic Zones (EEZs) to manage marine resources and protection, whose legal basis is established in The United Nations Convention on the Law of the Sea (UNCLOS) ^108^. Indeed, unlike the exploitation of hydrocarbons, the appropriation of wind resources requires states to create EEZs for wind energy ^109^, but the area's geography and the territories' proximity along the coasts result in overlapping claims over EEZs, leading to further state disputes. The seabed has increasingly become a contested space, with the risk of militarisation becoming the norm as well as growing threats to underwater infrastructure, as exemplified by the deployment of deep-operating drones by the French Navy ^110^. Ideally, joint management would benefit the international community. However, current legislation on the high seas is weak and non-binding, and each state has sovereign rights and environmental protection obligations within its EEZ. The Mediterranean Sea was historically excluded from maritime territorialisation, with coastal states limiting their claims to territorial seas and establishing fisheries and ecological protection zones until the 1990s ^111^. The discovery of offshore fossil energy deposits in the Mediterranean has highlighted the need for new boundaries, leading to efforts to establish EEZs ^112^.

Finally, raw material processing has a high environmental impact. New EU technologies could improve sector competitiveness and secure a sustainable supply of primary and secondary raw materials. Technological developments, such as iron nitride-based PM technology, which is cheaper and more abundant, are expected to be commercialised in the coming years, as well as the implementation of recycling techniques for materials already present. Adequate legislation and practices for these materials' collection and recycling capacity should be rapidly implemented in the EU. Labelling systems indicating the type of PM can help to improve the recycling process and achieve more efficient results. This would secure the magnet industries in the short term ^113^. However, heavy metal recycling technologies are still in their early stages of development and face many difficulties, such as the problematic separation of components, which makes the whole recycling process very costly, and the lack of information on the REEs content of different products. Technological advancements and improved recycling practices can reduce environmental impacts and enhance sustainability. However, efficient heavy metal recycling technologies and robust legislation are essential for long-term competitiveness and sustainability.

## Discussion and conclusion

Climate change is a major driver of biodiversity loss in the Mediterranean, affecting terrestrial and marine ecosystems. While substantial legislation exists to reduce greenhouse gas emissions and pollution on land and enhance environmental protection, the frameworks for safeguarding marine resources need to be updated to include recent technological developments in marine energy. This gap presents a significant challenge, as it risks prioritising climate change mitigation goals at the expense of marine ecosystems conservation. Consequently, achieving a Blue Economy that aligns with the GES may be challenging. OWFs are often championed for their role in decarbonisation, but their potential negative impacts on marine environments remain insufficiently assessed. Legislative inconsistencies further complicate this issue. As a result, the green transition carries significant costs, creating additional challenges in balancing environmental protection with renewable energy development.

In the Mediterranean, wind power is widely regarded as a sustainable solution to climate change. While wind energy is pollution-free and offers a long-term sustainable energy source, its uses may also cause irreversible damage to vulnerable marine ecosystems. The lack of sufficient research into the long-term effects of OWFs, coupled with gaps in existing legislation, impedes progress in reducing carbon footprints while safeguarding its unique marine environments. The region's distinctive characteristics, including its depth and environmental sensitivity, necessitate floating wind technology, a solution still in its early stages and not yet deployed at scale. The environmental impacts, feasibility, and long-term sustainability of floating wind farms remain poorly explored, underscoring the urgent need for comprehensive research and stronger regulatory frameworks before large-scale installations are implemented. Additional environmental concerns include the potential impact of seabed exploration and deep-sea mining, which could further threaten marine biodiversity. Addressing these issues requires a balanced approach, integrating renewable energy development with robust environmental protection measures to preserve the Mediterranean’s unique ecosystems for future generations.

The essay highlights the urgent need for legislative reform to address immediate challenges and develop a sustainable wind energy strategy in the Mediterranean. While offshore wind energy can reduce fossil fuel reliance, vague legislation coupled with limited research on socioeconomic and environmental impacts hinders progress. Despite efforts to protect maritime heritage, EU measures remain inadequate, requiring expanded protected areas and collaborative regional management. Moreover, diversifying the REE supply chain is critical to reducing EU dependence on China, achievable through partnerships and exploratory projects in northern EU countries like Sweden, Finland, Germany, and Norway ^114^. New studies on the manufacturing, operation, disposal, and recycling of wind turbines are needed, alongside international guidelines and pilot projects, to ensure sustainability and long-term viability. While offshore wind can contribute to sustainable development goals, its risks must be addressed through innovation, research, and a holistic regional approach. Otherwise, it may ultimately fall short of expectations as the "New Eldorado."

## Ethical approval and consent

Ethical approval and consent were not required.

## Data Availability

No data are associated with this article.
