# The Mediterranean Offshore Wind Turn: Lessons, Risks, and Regional Reflections

**DOI:** 10.12688/openreseurope.19339.2

**Published:** 2025-07-14

**Authors:** Marta Avesani

**Affiliations:** 1 EUROSUD Programme, University of Glasgow School of Social and Political Sciences, Glasgow, Scotland, G12 8RT, UK

**Keywords:** Mediterranean Sea, Green Deal, Barcelona Convention, Offshore Wind Energy, Floating Technology

## Abstract

The condition of the environment remains at the forefront of contemporary debates among political leaders and societal stakeholders, particularly in the context of addressing climate change. Offshore Wind Farms (OWFs) are emerging as a promising pathway for renewable energy development in the Mediterranean. While existing literature extensively explores the potential of OWF technology in advancing climate neutrality targets by 2030, comparatively little attention has been given to the challenges and trade-offs associated with its implementation. This essay examines the costs and benefits of deploying OWFs in the Mediterranean, assessing their economic, environmental, and social impacts through a trade-off analysis. Despite regulatory and environmental challenges, the deployment of OWFs in the Mediterranean is set to expand, driven by low marginal costs, favourable climatic and geographic conditions, and growing interest from international investors. The study underscores the urgent need for comprehensive legislation to address environmental, social, and economic risks, critically evaluating gaps in the current legal framework and examining the opportunities and challenges associated with renewable energy expansion. The analysis serves as a valuable resource for policymakers, investors, and environmental advocates, providing a framework to balance environmental preservation with the expansion of renewable energy. Addressing these critical considerations aims to support the creation of strategies that foster a harmonious integration of renewable energy projects within the Mediterranean region’s environmental and socio-economic landscape.

## Introduction

As the global transition becomes increasingly critical, debates and studies on renewables are gaining renewed attention. Seas and oceans act as vast collectors, primarily through solar power, which can be converted into potential, kinetic, and thermal forms. The Mediterranean Sea, capturing substantial solar energy and generating water currents, offers opportunities for power production via submerged turbines, transforming renewable and inexhaustible energy into electricity. Wind energy dominates the Mediterranean's renewable potential, especially with floating wind turbine technology advancements. However, despite existing measures in place, Europe’s natural environment is degrading rapidly, and it necessitates constant legislative updates to align urban and industrial growth with environmental sustainability ( Plan Bleu, 2022). In this respect, sustainable development focuses on fulfilling current needs while ensuring future generations can satisfy theirs ( Brundtland, 1987). It requires economic efficiency, social equity, and environmental sustainability while integrating ecological concerns with societal and financial priorities ( Delli Paoli & Addeo, 2019). Renewable energy derives from inexhaustible and continuously replenished sources, and it offers a crucial response to the climate crisis. In the Mediterranean, offshore wind energy, particularly through floating technology, presents a highly promising solution.

The European Union (EU) green transition has been shaped by several legislations and agreements, starting with the Renewable Energy Directive (RED) ( European Parliament and Council, 2023a), followed by RED II, which reinforced the promotion of renewable energy sources in line with updated sustainability goals ( European Parliament and Council, 2018). Most recently, Directive (EU) 2023/2413 updates RED II by setting more ambitious renewable energy targets and accelerating the energy transition ( European Parliament and Council, 2023a). In parallel, the EU’s long-term decarbonisation strategy was outlined in the Energy Roadmap 2050, published in 2011, which aimed to reduce greenhouse gas emissions by 80–95% by 2050 compared to 1990 levels ( European Commission, 2011). This vision was later integrated and strengthened under the European Green Deal and its Adaptation Strategy ( European Commission, 2019). The RED framework and its subsequent updates provide the legal foundation for the energy transition by removing barriers to renewable deployment, promoting renewable heating, cooling, and transport fuels, and supporting investment in cost-effective technologies ( Plan Bleu, 2022). The 2030 Energy and Climate Framework, initially set in 2014, aimed to reduce emissions by at least 40%, increase renewable energy use, and improve energy efficiency by 27% by 2030 ( European Commission, 2014). These targets have been strengthened under the Fit for 55 package to align with the EU's goal of reducing net greenhouse gas emissions by 55% by 2030 ( European Commission, 2023e). A 90% reduction in transport-related emissions is also needed, alongside a targeted increase in inland waterways and short-sea shipping by 25% by 2030 and 50% by 2050, though these measures may impact marine and coastal environments ( European Commission, 2019). More recently, the Clean Industrial Deal ( European Commission, 2025a) outlined a roadmap to boost industrial competitiveness and accelerate decarbonisation by expanding renewables, electrification, and circular economy practices by 2030. Renewable energy is central to cutting greenhouse gas emissions. Building on previous COP commitments, COP29 reaffirmed efforts to advance the Paris Agreement by accelerating energy system decarbonisation, expanding clean energy, phasing out fossil fuels, and aiming to limit global warming to well below 2°C, with efforts toward 1.5°C ( UNFCCC, 2025).

Wind energy is the EU’s second-largest source of power generation capacity, closely following gas installations ( deCastro *et al.*, 2019). Within this sector, offshore wind, implemented through offshore wind farms (OWFs), is expected to play an increasingly significant role in meeting the EU’s energy and climate objectives. Offshore turbines are installed at sea using foundation systems adapted to water depth and seabed conditions. Fixed-bottom foundations are typically suitable for shallow waters up to 50 metres, whereas floating platforms are required in deeper areas ( Nikitas *et al.*, 2019). Offshore wind offers several advantages over onshore systems, including exposure to stronger, more consistent wind conditions, which improves generation efficiency ( Bilgili *et al.*, 2011). The Mediterranean presents a distinct opportunity for offshore wind development. Although there are currently no operational OWFs in the region ( Plan Bleu, 2022), growing interest has been driven by favourable wind resources, declining technology costs, and increased international attention. Due to the prevalence of deep waters, fixed-bottom foundations are generally not feasible, making floating wind the most suitable approach ( Martinez & Iglesias, 2021). Floating turbines can access up to 80 per cent of global offshore wind resources located in waters deeper than 50 metres ( WindEurope, 2023), and may offer additional benefits such as reduced visual impact and less interference with maritime and fishing activities ( Farr *et al.*, 2021; Le Visage, 2022). Recognising this potential, the European Commission launched a strategy in 2020 to expand marine renewable energy, setting targets to increase offshore wind capacity from 12 gigawatts to 60 gigawatts by 2030, and to 300 gigawatts by 2050 ( European Commission, 2020a). In October 2023, a revised strategy raised the 2030 target to 111 gigawatts and introduced measures to streamline permitting, enhance regional cooperation, and strengthen supply chains ( European Commission, 2023c). In addition to supporting climate goals, the expansion of offshore wind has the potential to reduce fossil fuel imports, improve air quality, and mitigate climate-related risks ( WindEurope, 2023).

Achieving these objectives will require coordinated action across Member States, including the development of national low-carbon transition plans. In France, several floating wind farms are under development in the Golfe du Lion off the southern coast (Occitanie region), notably the “Éoliennes Flottantes du Golfe du Lion” (EFGL) project, which comprises pilot and pre-commercial sites along with two additional pilot projects ( Gouty, 2022). Similarly, Italy is actively advancing floating offshore wind projects, particularly around Sicily, Sardinia, and Apulia, with government-supported initiatives reflecting a strategic emphasis on floating technology ( Watson Farley & Williams, 2025). Spain’s offshore wind activities have historically focused on the Atlantic coast, where a mixture of fixed-bottom and floating farms exist; however, recent efforts on the Mediterranean coasts, specifically in the Balearic and Catalan regions, are concentrating on floating wind pilot projects to address the challenges posed by deeper waters, where Spain is developing the “Parc Tramuntana” Floating Offshore Wind Farm Project ( Diez-Caballero *et al.*, 2022). Greece has also recently enacted legislation to facilitate offshore wind development, with maritime spatial planning underway to identify suitable zones predominantly for floating wind farms in the Aegean, Ionian, and certain Mediterranean-facing coastal areas ( Alma Economics, 2021). These initiatives align with national plans outlined in the Maritime Spatial Planning Directive amendment 2023 ( European Parliament and Council, 2023b).

However, given the unique characteristics of the Mediterranean Sea, evaluations of potential impacts remain speculative due to the lack of concrete operational examples. Research focuses on OWFs in the North, Baltic, and North Atlantic, which differ from the Mediterranean’s diverse shelf morphology and fragile benthic habitats like seagrass meadows and coralligenous reefs ( Lloret *et al.*, 2022). The limited and fragmented nature of existing research on offshore wind development in the Mediterranean has fostered an overly optimistic view of its contribution to the green transition, often overlooking region-specific risks. Much of the current literature prioritises technical feasibility and economic potential, while long-term environmental and socio-economic implications remain underexplored. In particular, the impacts of deep-water floating infrastructure, now central to Mediterranean deployment plans, are insufficiently understood. Concerns include pollutant release from turbine materials, electromagnetic fields from subsea cabling, and underwater noise, alongside broader disruptions to marine ecosystems and coastal livelihoods. While these issues have been studied in Northern European contexts, Mediterranean-specific conditions, such as its semi-enclosed geography, high biodiversity, and densely populated coastlines, necessitate a more tailored analysis. This paper first examines the legislative framework governing offshore wind at EU level, highlighting its limited applicability in the Mediterranean and identifying key regulatory and enforcement shortcomings. It then assesses the environmental, social, and economic challenges associated with offshore wind deployment in the region, drawing on recent studies and pilot projects. The findings suggest that without integrated, context-specific governance, the benefits of offshore wind may be undermined by unaddressed harms, highlighting the need to align decarbonisation with strong environmental and social protections.

## Marine environmental governance and policy frameworks in the Mediterranean

Since the 1992 Rio Declaration on Environment and Development ( United Nations, 1992), the international community has intensified efforts to promote the sustainable use of marine resources. The Declaration remains a foundational legal and policy instrument, having established core principles, such as the precautionary principle, polluter pays, and common but differentiated responsibilities, that continue to shape global and regional environmental governance frameworks today. A notable advancement in this regard was the adoption of Sustainable Development Goal 14 in 2015, which seeks to “conserve and sustainably use the oceans, seas, and marine resources for sustainable development” ( United Nations General Assembly, 2016). In parallel with these global commitments, the Mediterranean Action Plan (MAP) was instituted in 1975 as a multilateral environmental agreement under the auspices of the United Nations Environment Programme (UNEP). Its principal aim has been the protection of the marine environment through a harmonised regional framework. Over subsequent decades, UNEP/MAP has evolved into a model of global governance, instrumental in the negotiation and adoption of the Convention for the Protection of the Marine Environment and the Coastal Region of the Mediterranean (Barcelona Convention) and its related protocols. With seven protocols under the MAP, the Barcelona Convention is the region’s key legally binding multilateral environmental agreement ( UNEP/MAP, 1995), which came into force in 2004. Its Contracting Parties (CP) consist of 21 Mediterranean countries and the EU, encompassing the Mediterranean Sea eastward from the Strait of Gibraltar. The Convention allows each party to extend its application to its national territory, ensuring a broad and adaptable scope for regional cooperation.

Cooperation mechanisms, such as those under the Barcelona Convention, aim to coordinate the use of maritime resources, strengthen governance, and foster economic growth while promoting sustainable practices ( Plan Bleu, 2022). These efforts align with broader frameworks of renewable energy policies and marine ecosystem protections. According to Art.4, the CPs agree to take all necessary measures, individually or jointly, to prevent, reduce, and eliminate pollution of the Mediterranean Sea and to protect the marine environment to support sustainable development ( UNEP/MAP, 1995). A key component is the Dumping Protocol, entered into force in 1978, and amended in 1995 ( PNUE/PAM, 1976). Excluding Montenegro, all CPs to the Barcelona Convention are also parties to this protocol, which prohibits dumping activities, incineration at sea, and waste disposal, except for specific materials ( PNUE/PAM, 1976). In 2021, during the 22nd Meeting of the CPs in Turkey, a decision was made to amend the Annex to the Dumping Protocol. These amendments, binding in the EU under Art. 29 of the Barcelona Convention, revised the criteria for issuing permits for disposal at sea. The updated criteria outline the characteristics and composition of dumped materials and the attributes of dumping sites and deposit methods ( European Commission, 2021b). This reflects the CPs’ commitment to reducing pollution and ensuring sustainable management of the Mediterranean Sea.

In the Mediterranean, efforts are guided by the Mediterranean Strategy for Sustainable Development (MSSD), adopted during COP19 of the Barcelona Convention in 2016. The MSSD provides a framework to integrate regional, sub-regional, and national policies with the 2030 Agenda for Sustainable Development ( Mancini, 2022). This approach aligns with the EU Marine Strategy Framework Directive (MSFD) 2016–2025, which states that ecosystem protection is a cornerstone of economic and environmental policy ( European Commission, 2020b). It builds on this by targeting the Good Environmental Status (GES) of marine and coastal environments. The MSFD fosters ecosystem-based management, harmonising efforts among 23 coastal and five landlocked EU states and coordinating with non-EU countries to sustainably manage a 5.72 million km ^2^ marine area ( European Commission, 2020b). In 2018, the CPs of the Barcelona Convention adopted the Ecosystem Approach (EcAp) and agreed on a roadmap for its implementation in the Mediterranean. The EcAp is a strategy for the integrated management of land, water, and living resources that promotes their conservation and equitable use. As such, it is the guiding principle for policy development and implementation undertaken under the auspices of the Barcelona Convention, such as achieving GES in the Mediterranean marine and coastal environment through informed management decisions based on integrated quantitative assessment and monitoring ( SPA/RAC, 2015). At the Mediterranean level, the EcAp and the MSFD are implemented in synergy and coherence, and they are among the most ambitious international frameworks for marine environmental protection ( European Commission, 2025b). Additional measures include Art.9 of the Integrated Coastal Zone Management Protocol (ICZM) and the Union for the Mediterranean’s 2015 Ministerial Declaration, all of which emphasise ecosystem preservation. EU policies, such as the Water Framework Directive (WFD) and its updates, further reinforce these efforts by aiming to achieve good ecological status for water bodies. Despite delays from funding and implementation challenges, the WFD created a governance framework for integrated water management, reducing chemical pollution and connecting human activities to environmental impacts ( European Parliament and Council, 2000).

This integrated approach ensures marine ecosystems remain functional and resilient for future generations. After launching the world’s first global framework to finance a sustainable Ocean Economy ( European Investment Bank, 2018), the EU introduced its new strategy on Biodiversity for 2030, focusing on “making nature healthy again” ( Bassetti, 2021). The strategy boosts the protection of marine ecosystems by expanding the protected areas and stressing the importance of the EcAp for managing human activities at sea. Sustainable innovation presents significant opportunities in green ports, shipping, aquaculture, water management, conservation, recycling, and maritime ecotourism, all key to the region's future. Meanwhile, the EU Commission emphasised the benefits of sustainable blue economy activities, advocating for an international Blue Economy Sustainability Framework ( European Commission, 2021c). However, according to the results of the 2020 report on the first implementation cycles of the MSFD, the EU framework for protecting the marine environment needs to be strengthened ( European Commission, 2020a). Indeed, GES is achievable only if the pressures on the marine and coastal environment decrease and, therefore, if the uses of the marine and coastal environment that cause these pressures are adjusted. Surprisingly, the notion of GES appears only once in the text of the European Green Deal and only once in the Commission’s Communication on the Sustainable Blue Economy ( European Commission, 2021a). This may indicate a persistent lack of integration of marine protection into broader policies or a dominance of economic priorities over marine conservation, even under the guise of climate change adaptation.

To address environmental challenges, the EU approved a taxonomy defining criteria for economic activities contributing to climate adaptation and prioritising adaptation solutions ( European Commission, n.d.). Recognising the scale and diversity of its natural heritage, the EU has developed extensive legislation to protect and enhance Mediterranean biodiversity. Central to this is the Nature Directives, which establish Natura 2000, a network of protected areas comprising Special Areas of Conservation and Special Protection Areas under the Habitats Directive ( Council of the European Communities, 1992) and the Birds Directive ( European Parliament and Council, 2009). Natura 2000 ensures the conservation of rare, threatened, or endemic species, including all wild bird species, through protection, management, and monitoring measures. It covers 18% of the EU’s land area and 6% of its sea area, although significant gaps remain in marine coverage, and only half of the Natura sites have management plans with conservation objectives ( European Commission, 2014). The “Fitness Check” evaluation confirmed the directives’ effectiveness but emphasised the importance of practical implementation. Since then, legislative advancements have enhanced marine protection within the Natura 2000 framework. In 2023, Implementing Decision (EU) 2023/2806 standardized site-specific data formats, improving transparency in reporting across Member States ( European Commission, 2023b). Additionally, the Nature Restoration Law, adopted in June 2024, introduced legally binding restoration targets aiming to restore at least 20% of the EU’s land and marine ecosystems by 2030, focusing on Natura 2000 sites, and up to 90% of degraded habitats by 2050 ( European Parliament, 2024). These initiatives are complemented by the ongoing revision of the Marine Strategy Framework Directive to strengthen the assessment and achievement of Good Environmental Status ( European Commission, 2025b).

## Legal context of offshore wind in the Mediterranean

In the Mediterranean, OWFs are vital to the Blue Economy, a sector generating significant regional income. As the offshore wind industry expands, its environmental impacts, particularly on marine ecosystems, remain insufficiently studied. The sector has evolved from utilising fixed-bottom turbines in shallow waters up to 60 m deep to deploying floating turbines in depths exceeding 120 m, with current technological developments exploring installation in waters as deep as 1,000 m ( Farr *et al.*, 2021). The Blue Economy encompasses traditional maritime activities such as fishing, aquaculture, coastal tourism and emerging sectors such as renewable energy and marine biotechnologies ( European Commission, 2021a). The Sustainable Blue Economy focuses on clean technologies, renewable energy, and circular material flows to secure long-term social and economic stability within planetary boundaries ( World Wide Fund, 2015). It seeks to balance the optimal use of maritime natural capital with human and social capital, advancing sustainable development, social equity, and reduced ecological risks. The EU's taxonomy for climate change mitigation, outlined in Regulation (EU) 2020/852, provides a framework for determining whether economic activities and investments qualify as environmentally sustainable, including provisions for the marine environment ( European Parliament and Council of the European Union, 2020). The regulation focuses on transitioning to renewable energy, reducing fossil fuel reliance, and promoting carbon-neutral energy sources. It allows activities with no viable low-carbon alternatives as long as they limit global temperature rise to 1.5°C above pre-industrial levels. Such activities must comply with strict GHG emissions standards and support the development of low-carbon alternatives. In June 2023, the Commission adopted delegated acts expanding the Taxonomy Regulation beyond climate objectives to include technical screening criteria for four additional environmental goals, including the sustainable use and protection of water and marine resources ( European Commission, 2023d).

Commission Delegated Regulation (EU) 2023/2485 establishes the general framework for determining whether an economic activity and its investments qualify as environmentally sustainable and includes several chapters related to the marine environment ( European Commission, 2023a). It ensures the conservation of rare, threatened, and endemic species, including all wild bird species in the EU, through measures for their protection, management, and monitoring. It also highlights the restoration of wetlands and the development of renewable energy sources such as wind, ocean, hydropower, geothermal energy, and cogeneration technologies. Furthermore, it states that offshore wind projects must not hinder achieving GES as defined by the MSFD, requiring measures to prevent or mitigate impacts on biodiversity and seabed integrity, supported by an environmental impact assessment and necessary mitigation and compensation measures. However, the references to the proper use of offshore wind turbines contained in the Regulation are insufficient to address their wide-ranging environmental impacts, including harm to marine ecosystems and pollutant emissions during their creation and dismantling. Furthermore, their buoys or mooring systems could contain one or more environmentally hazardous substances.

Natura 2000 sites, including Special Protection Areas for birds, are among the EU’s most biodiverse and valuable areas and must be protected from OWFs developments. Damage to these sites risks their legal status and may result in condemnation of member states ( Gouty, 2021). Although the Habitats Directive does not outright prohibit wind farms within Natura 2000 sites, it requires a case-by-case assessment demonstrating that the project will not cause significant adverse impacts, such as habitat loss, degradation, fragmentation, species disturbance, or other indirect effects on environmental quality ( Fabrégat, 2021). Such assessments must consider the scale and magnitude of impacts and ongoing projects in the area. When appropriately integrated with other economic activities, including other renewable energy sources, the negative impacts of OWFs could be reduced, and biodiversity may even benefit; however, there is no current legal basis at the national or EU level to guarantee this outcome. The European Commission’s guidance document on wind energy developments in Natura 2000 sites aims to align with updated EU renewable energy and offshore wind policies ( European Commission, Directorate-General for Environment, 2020). It clarifies the application of Natura Directives and updates the 2011 guidance on wind farms in protected areas, emphasising the need for further knowledge, best practices, and policy integration. Nonetheless, as EC communications are non-legally binding ( EUR-Lex, 2007), this guidance serves primarily an informational role and lacks enforceability ( European Parliament and Council of the European Union, 2020). This limits its credibility and legal weight, underscoring the necessity for thorough case-by-case analyses prior to OWF development and revealing gaps in EU legislation where advances in renewable energy may conflict with biodiversity protection goals.

## Environmental challenges of offshore wind farms: Differentiating fixed and floating technologies

Although OWFs contribute significantly to climate change mitigation by reducing greenhouse gas emissions, both fixed and floating systems introduce distinct environmental risks that need to be considered throughout the entire lifecycle, from raw material extraction and construction to operation, maintenance, and decommissioning ( Garcia-Teruel *et al.*, 2022). As shown in Table 1, both fixed and floating offshore wind technologies pose various environmental challenges, although their impacts differ depending on location, seabed disturbance, and marine biodiversity. The synthesis is based on a range of recent studies (see Table 1 for an overview).

**Table 1.** Environmental Challenges of Offshore Wind Farms - Fixed vs. Floating. Summary of key environmental risks associated with fixed-bottom and floating offshore wind technologies. Source: Author's own synthesis based on existing literature.

FLOATING OFFSHORE WIND FARMS	FIXED (ANCHORED) OFFSHORE WIND FARMS	CROSS-CUTTING CONCERNS
• Deployed in deeper offshore watters (>120 m to ∼1000 m), using moored floating platforms • Seabed disturbance from anchoring systems in deep-sea habitats • Wider operational footprint, with greater towing frequency (increased Spill and emission risk) • Extended subsea cabling, resulting in broader EMF/thermal impacts and larger exclusion zones • Deployment in largely undisturbed marine areas, with uncertain long- term ecological effects • Amplified artificial reef effects, including enhanced biodiversity but higher invasive species risk	• Deployed in shallow coastal waters (≤60 m), often ecologically sensitive • Installed in shallow, biodiverse coastal areas • Habitat disturbance (Maerl beds, Posidonia oceanica) • Bird & bat collision risk (migratory & nocturnal species) • Underwater noise pollution (pile driving) • Pollution from construction (toxic metals, sediment remobilization) • EMF & thermal emissions from power cables • Artificial lighting effects on nocturnal species • Risk of accidental pollution (spills, fires) • Artificial reef effect (biodiversity + invasive species risk) • Marine reserve effect (restricted fishing benefits and risks) • Altered oceanographic processes (currents, turbulence)	• Biodiversity loss • Underwater acoustic pollution • Chemical and electromagnetic pollution **Policy & Mitigation Measures** • Apply the Precautionary Principle and ensure comprehensive environmental assessments • Implement real-time ecological monitoring (e.g. radar, camera systems) • Schedule seasonal shutdowns during peak migratory periods • Use turbine blade modifications, such as UV-reflective paint, to reduce bird strikes • Deploy acoustic deterrents to protect marine mammals and fish • Promote long-term ecological studies, particularly on floating wind impacts

### Environmental impacts of fixed offshore wind farms

Fixed-bottom offshore wind farms are typically installed in shallow, coastal areas, which are often rich in marine biodiversity. One of the primary environmental challenges (1) is the disturbance and degradation of sensitive habitats such as Maerl beds and seagrass meadows, notably those formed by *Posidonia oceanica*, a protected and ecologically important species ( Barberá *et al.*, 2003; Telesca *et al.*, 2015). These ecosystems provide essential shelter, nursery areas, and carbon sequestration functions, and their disruption can lead to a cascade of ecological consequences.

(2) The risk of bird and bat collisions with turbine blades is a significant concern, especially for migratory species. Seabirds in the Mediterranean region, many of which are endangered, may have their migration routes altered or interrupted due to the placement of turbines ( Conseil national de la protection de la nature, 2021; UNEP/MAP, 2022). In addition, nocturnal species such as bats are vulnerable to direct collision and displacement effects.

(3) Underwater noise generated during construction, particularly from pile driving, represents a significant source of acoustic pollution. Marine mammals, which rely heavily on sound for navigation, communication, and foraging, are especially susceptible to noise disturbance, which may result in physical injury or long-term behavioural changes ( Carrillo, 2010; Solangi, 2019).

(4) Pollution from construction and operational activities, including the leaching of toxic metals from sacrificial anodes, poses another significant threat. These anodes, used to protect submerged infrastructure, can release heavy metals into the marine environment, forming toxic compounds and degrading water quality ( Kirchgeorg *et al.*, 2018). Similarly, the remobilisation of sediments during geotechnical work can release trapped nutrients or pollutants, further impacting water column chemistry and benthic communities.

(5) The electromagnetic fields (EMFs) and thermal emissions from underwater power cables used to transmit electricity to shore can disrupt species sensitive to electric and magnetic cues. Fish, elasmobranchs, and other organisms that rely on geomagnetic navigation or electroreception may experience disorientation and altered behaviour ( Donázar-Aramendía *et al.*, 2025; Emeana *et al.*, 2016).

(6) Artificial lighting associated with OWF construction, maintenance, and nighttime operation presents a visual disturbance that can disorient nocturnal marine species such as seabirds and sea turtles. These lights attract animals to turbine sites, increasing the risk of collision and altering natural movement patterns ( Defingou *et al.*, 2019)

(7) Accidental events such as ship collisions, fires, or extreme weather events can lead to chemical or oil spills, introducing acute pollution risks to marine ecosystems. These events highlight the importance of risk management and emergency response planning at OWF sites ( Arisian *et al.*, 2017).

(8) Introducing artificial substrates through turbine foundations may lead to the formation of artificial reefs, generating what is known as the “reef effect.” This phenomenon can benefit local biodiversity by attracting marine species in otherwise homogenous sandy habitats; however, it may also facilitate the spread of invasive species and lead to the exclusion of some native or mobile species ( Danish Energy Agency, 2006; Plan Bleu, 2022).

(9) Fixed OWFs can act as de facto marine reserves due to restricted fishing activity in their vicinity. This “reserve effect” may enhance local fish stocks, but overfishing at the edges of these zones may reduce or negate broader conservation benefits, limiting the so-called “spillover effect” ( Lacroix & Pioch, 2011).

(10) Finally, OWFs influence local oceanographic processes. Turbine structures can modify current patterns, induce upwelling or downwelling, and alter turbulence and bathymetry. These changes can have cumulative ecological effects, mainly when multiple farms are clustered together ( Durning *et al.*, 2020; Lindeboom *et al.*, 2015).

### Environmental impacts of floating offshore wind farms

Floating offshore wind technology offers several environmental advantages compared to fixed systems. These include reduced visual impact due to deployment farther from shore and avoidance of shallow coastal habitats. However, floating systems also present their own set of environmental concerns.

(1) Mooring and anchoring systems, such as suction piles or drag-embedded anchors, can disturb previously undisturbed deep-sea habitats. These habitats are often poorly understood and may harbor fragile ecosystems susceptible to mechanical disruption ( Garcia-Teruel *et al.*, 2022).

(2) The operational footprint of floating turbines can be larger than that of fixed systems, particularly because floating platforms may need to be towed to shore for major maintenance. This increases the frequency and scale of marine operations and raises risks of spills, emissions, or ballast water discharges ( Mendecka & Lombardi, 2019).

(3) The need for longer and more complex subsea cable systems introduces greater cumulative EMF and thermal emissions, which may affect wider swaths of benthic and pelagic environments. These cables also contribute to excluding fishing activities over large marine areas ( Le Visage, 2022).

(4) Floating wind farms are increasingly being planned for deployment in deep offshore regions that have, until now, experienced limited industrial activity. Consequently, the environmental impacts in these areas are more difficult to predict, and there is a greater need for precautionary governance ( Environmental Protection Authority Western Australia, 2016).

(5) As with fixed systems, floating OWFs may generate artificial reef effects and facilitate the spread of non-native species. However, their increased mobility and deeper location could exacerbate these dynamics in less resilient ecosystems.

## Socio-economic challenges

Limited research and monitoring exist regarding the socio-economic impacts of OWFs in the Mediterranean, where such considerations are frequently overshadowed by environmental and technical-economic concerns. The cumulative socio-economic impacts encompass effects on employment, economic growth, housing, local services, community well-being, and conflicts with established sectors such as tourism and fisheries. From an economic standpoint, OWFs influence employment, gross value added, and various coastal industries including fishing, shipping, and recreation. The construction and maintenance phases generate employment opportunities, often supported by training programmes and initiatives to develop local supply chains. These projects also contribute to infrastructure improvements, particularly with regard to ports, delivering socio-economic benefits during the development phase. Nevertheless, the social impacts can include increased pressure on housing and overall quality of life in coastal communities. In some cases, developers offer housing assistance or mitigation measures to address these challenges. Tourism, an essential economic driver in the Mediterranean, especially in relation to coastal and marine recreational activities such as sailing, diving, swimming, and wildlife observation, may be adversely affected by the presence of OWFs. These developments can restrict access to key areas and alter the visual seascape, potentially leading to significant reductions in tourism revenues ( Lloret *et al.*, 2022).

Given the narrow continental shelves of many Mediterranean coastal states, OWFs are often situated relatively close to shore. This proximity may negatively affect the visual character of the seascape, deterring tourists and raising concerns about potential economic losses within affected communities ( Westerberg *et al.*, 2011). In response, Community Benefit Agreements have been introduced to acknowledge the involvement of local populations. These are offered not as compensation, but as a token of appreciation for supporting projects deemed in the national interest ( Durning *et al.*, 2020). Nevertheless, community acceptance remains essential, as residents may view OWFs as symbols of marine industrialisation, reinforcing broader economic anxieties.

The exclusion of fishing activities from OWF zones presents a significant threat to small-scale fisheries, which are prevalent across Mediterranean coastal regions. According to Chaji & Werner (2023), offshore wind developments present several economic challenges for the fishing industry. Key concerns include increased fuel expenditure due to the displacement of fishing activities, reduced income and employment stability, elevated insurance costs associated with navigational hazards near wind farms, and adverse impacts on ancillary fishing businesses. The exclusion of fishing from OWF zones, particularly during the construction phase, poses a considerable threat to small-scale fisheries, which are especially prevalent along Mediterranean coastlines. Moreover, marine disturbances caused by installation and operational activities may diminish fishery productivity, thereby exacerbating existing economic vulnerabilities within the sector. Moreover, these restrictions can hinder scientific fishery surveys, which are vital for long-term ecosystem monitoring. Conversely, exclusion zones around OWFs may function as de facto marine reserves, contributing to biodiversity conservation. Although buffer zones between OWFs and Marine Protected Areas may provide partial protection, further research is necessary to evaluate their effectiveness. Critically, the most technically viable sites for OWF deployment often coincide with MPAs due to favourable wind and bathymetric conditions. This creates a risk that energy companies may prioritise technical feasibility over ecological sensitivity. The Mediterranean’s status as one of the world’s most heavily trafficked seas further complicates OWF deployment. The installation of wind farms may reduce navigable space, increase collision risks for commercial and passenger vessels, and redirect traffic onto already congested routes ( Le Visage, 2022).

The generation of wind energy depends on materials associated with high supply risks. Modern turbines employ advanced permanent magnet (PM) generators and lightweight, fatigue-resistant blades designed for high efficiency and durability. Turbine components are typically constructed from steel alloys incorporating nickel, manganese, titanium, and other elements, while blades are composed of fibreglass and resin. Critically, the production of these technologies is heavily reliant on rare earth elements (REEs) such as neodymium and dysprosium, resources that are largely imported from outside the EU with China and Latin America accounting for the majority of supply ( Bobba *et al.*, 2020). Demand for these materials is projected to increase substantially by 2050 ( Hund *et al.*, 2020). Although the processing of raw materials remains environmentally intensive, emerging technologies offer opportunities to enhance both competitiveness and sustainability. For instance, iron nitride-based PMs, which utilise more abundant materials and offer cost advantages, are expected to become commercially viable in the near future. Similarly, advancements in recycling techniques for REEs could substantially reduce environmental impacts. While recycling REEs remains challenging at every stage, recovering them from consumer materials presents a promising alternative to conventional extraction methods ( Balaram, 2019). However, to capitalise on these developments, the EU must implement robust legislation to support material collection, recycling infrastructure, and transparent labelling systems for PM content ( Hund *et al.*, 2020). Despite progress, complex component separation and limited data on REE content in end-of-life products make recycling costly and inefficient. Continued innovation, policy support, and industry collaboration are essential for sustainable material cycles.

This dependency raises concerns about the EU’s strategic autonomy and the long-term viability of its energy transition. Although the cost of renewable energy is decreasing, the extraction and processing of raw materials pose significant environmental challenges. The mining sector currently accounts for between 2% and 11% of global energy consumption ( Guilbaud, 2016). Climate-smart mining practices are necessary to prevent shifting emissions from power and unsustainable extraction could undermine the environmental benefits of wind energy. Climate-smart mining practices will be essential to prevent the transfer of emissions from energy production to raw material sourcing. Moreover, wind turbine costs are highly sensitive to fluctuations in metal prices, particularly those linked to REEs. The supply chain for wind technology is vulnerable at various stages, from raw material sourcing to component manufacturing. China’s near-monopoly on REEs and PMs remains a strategic concern, exemplified by the 2011 market restrictions that triggered global price volatility ( Bobba *et al.*, 2020).

In response, several European states have accelerated seabed exploration to identify domestic sources of REEs. Polymetallic nodules and cobalt-rich crusts in deep-sea environments are emerging as key sources of critical materials, drawing growing interest due to their vast metal content and mining potential ( Hund *et al.*, 2020). While seabed mapping aligns with UNESCO’s target of charting 80% of the ocean floor by the end of the decade, it often precedes commercial exploitation. Deep-sea mining raises serious environmental concerns, including sediment disturbance, noise, light pollution, and the introduction of invasive species ( United Nations Environment Programme, 2014). Exploring deep seabeds is often followed by exploiting these areas, which are still mysterious and widely unknown. However, deep-sea mining presents environmental threats. Extraction involves pulverising mineral crusts pumped to vessels, releasing pollutants, noise, light, and invasive species. The light affects deep-water species, while the altered water composition reduces oxygen and clouds the environment ( United Nations Environment Programme, 2014). These activities may significantly alter marine ecosystems, reduce oxygen levels, and impair water clarity. Deep-sea vent communities are especially vulnerable and can take years to recover from disturbance ( Joseph, 2017; Van Dover, 2011).

The strategic implications of OWF development are increasingly evident in the Mediterranean, where coastal states are asserting claims to marine resources through the establishment of Exclusive Economic Zones (EEZs), in accordance with the United Nations Convention on the Law of the Sea (UNCLOS) ( United Nations, 1982). Unlike hydrocarbon exploitation, wind energy development requires similar territorial claims, which has led to overlapping EEZs and escalating disputes due to the region’s complex geography ( Le Visage, 2022). The seabed has become a contested space, with increasing militarisation and concerns over the security of underwater infrastructure, as evidenced by the deployment of deep-operating drones by the French Navy ( Kloetzli, 2022). Despite these tensions, joint management and cooperation would benefit regional stability and environmental protection. Historically, Mediterranean states limited maritime claims to territorial seas and ecological protection zones. However, the discovery of fossil fuel deposits and the advancement of renewable energy have prompted a shift towards full EEZ declarations ( Le Visage, 2022; Pellen-Blin *et al.*, 2019).

Concurrently, the offshore wind sector is progressing beyond the demonstration stage. However, large-scale deployment is constrained by supply chain bottlenecks, insufficient port infrastructure, logistical challenges, and a lack of specialised installation vessels. As turbines increase in size and are sited further offshore, these barriers become more pronounced ( Torres *et al.*, 2023). The construction process is also weather-dependent, requiring uninterrupted operations and substantial storage facilities. High vessel rental costs and port constraints pose further difficulties ( Vis & Ursavas, 2016). Therefore, addressing component availability, infrastructure limitations, and logistical capacity will be essential to enabling the sector’s future growth.

## Discussion and conclusion

Climate change is a principal driver of biodiversity loss in the Mediterranean, affecting both terrestrial and marine ecosystems. While substantial legal frameworks exist to reduce land-based greenhouse gas emissions and environmental degradation, marine governance remains fragmented and poorly aligned with recent technological advancements in marine renewable energy. This regulatory gap risks prioritising climate mitigation at the expense of marine conservation, thereby undermining the region’s ability to GES and a genuinely sustainable Blue Economy. OWFs are often promoted as key instruments of the green transition, yet their environmental and socio-economic impacts, particularly in the Mediterranean, remain underexplored ( Defingou *et al.*, 2019; Plan Bleu, 2022). Technological innovations such as radar and camera systems for monitoring bird movements, ultraviolet-reflective turbine blades, acoustic deterrents, and temporal shutdowns during migratory periods may reduce ecological harm. However, these must be supported by rigorous ecological baselines, real-time monitoring, and long-term studies, especially as floating wind technologies are deployed at increasing depths and scales. The region’s deep waters and ecological sensitivity necessitate floating wind technology, a solution still in its early stages and not yet deployed at scale. While wind energy is generally regarded as a clean alternative to fossil fuels, the construction and operation of OWFs, both fixed and floating, pose significant risks including underwater noise, habitat disturbance, chemical and electromagnetic pollution, and cumulative impacts from multiple installations. These threats are particularly acute in biodiversity-rich or poorly studied marine areas.

Current legislative frameworks exhibit inconsistencies and gaps that hinder effective risk assessment and mitigation. The lack of harmonisation across EU directives and limited coordination among Member States exacerbate these challenges. Comprehensive environmental impact assessments must be mandated for all project stages, from site selection through to decommissioning, integrating cumulative and synergistic impacts. The application of the Precautionary Principle, embedded in the Treaty on the Functioning of the European Union and reflected in the Marine Strategy Framework Directive and the Maritime Spatial Planning Directive, is essential to safeguard marine biodiversity ( European Parliament and Council, 2023b). The findings highlight the urgent need for legislative reform and stronger regulatory frameworks to support a sustainable offshore wind strategy in the Mediterranean. Inadequate legislation and limited research on socio-economic and environmental impacts currently impede progress. Protection of maritime cultural heritage and biodiversity requires expanded protected areas and enhanced regional cooperation. Furthermore, diversifying the REEs supply chain is critical to reducing EU dependence on China and can be advanced through partnerships and exploratory projects in northern European countries such as Sweden, Finland, Germany, and Norway ( Bobba *et al.*, 2020).

Offshore wind energy offers significant potential to contribute to decarbonisation and sustainable development goals. Yet, without a more holistic, evidence-based, and precautionary approach, the sector risks replicating environmental harms it seeks to mitigate. The Mediterranean’s energy future depends not only on technological innovation but also on robust governance, environmental integrity, and collaborative regional management. More rigorous ecological baselines and long-term studies are urgently needed as offshore wind development expands into deeper waters through floating technologies. Policy frameworks must maintain environmental integrity alongside energy transition goals, particularly in biodiversity-rich or understudied marine regions. New studies on the manufacturing, operation, disposal, and recycling of wind turbines are needed, alongside international guidelines and pilot projects, to ensure sustainability and long-term viability. While offshore wind can contribute to sustainable development goals, its risks must be addressed through innovation, research, and a holistic regional approach. Otherwise, it may ultimately fall short of expectations as the “New Eldorado”.

## Ethical approval and consent

Ethical approval and consent were not required.

## Data Availability

No data are associated with this article. The table is based on the author’s synthesis of peer-reviewed literature and does not rely on a primary dataset. No external data are associated with this table.
