# The Mediterranean Offshore Wind Turn: Lessons, Risks, and Regional Reflections

**DOI:** 10.12688/openreseurope.19339.3

**Published:** 2025-10-22

**Authors:** Marta Avesani

**Affiliations:** 1 EUROSUD Programme, University of Glasgow School of Social and Political Sciences, Glasgow, Scotland, G12 8RT, UK

**Keywords:** Mediterranean Sea, Green Deal, Barcelona Convention, Offshore Wind Energy, Floating Technology

## Abstract

The condition of the environment remains at the forefront of contemporary debates among political leaders and societal stakeholders, particularly in the context of addressing climate change. Offshore wind farms are emerging as a promising pathway for renewable energy development in the Mediterranean. While existing literature extensively explores the potential of offshore wind technology in advancing climate neutrality targets by 2030, comparatively less attention has been paid to the challenges and risks of its implementation. This essay examines the economic, environmental, and social dimensions of offshore wind farm deployment in the Mediterranean, highlighting both its benefits and associated risks. Despite regulatory and environmental obstacles, expansion is expected, driven by low marginal costs, favourable conditions, and growing international investment. The study identifies critical gaps in the current legal and governance frameworks and evaluates their capacity to manage environmental and socio-economic impacts. It also provides insights into strategies and policy measures that could enhance the sustainable integration of offshore wind energy, supporting informed decision-making for policymakers, investors, and environmental stakeholders in the Mediterranean region.

## Introduction

As the global transition to clean energy becomes increasingly urgent, renewable energy sources are receiving renewed attention in both research and policy debates. Seas and oceans function as vast energy collectors, primarily through solar radiation, which can be harnessed in potential, kinetic, and thermal forms ( Bilgili *et al.*, 2011). The Mediterranean Sea, with its high solar exposure and dynamic water currents, presents opportunities for energy generation via submerged turbines that convert renewable, inexhaustible energy into electricity. Wind energy, particularly through advancements in floating turbine technology, holds the greatest renewable potential in the region ( Plan Bleu, 2022). However, despite existing measures, Europe’s natural environment continues to degrade rapidly, highlighting the need for continuous legislative adaptation to balance urban and industrial development with ecological preservation. In this context, sustainable development aims to meet present needs without compromising the ability of future generations to meet theirs ( Brundtland, 1987), requiring the integration of economic efficiency, social equity, and environmental stewardship ( Delli Paoli & Addeo, 2019). Offshore wind, especially floating technologies in the Mediterranean, stands out as a key pathway toward aligning energy production with long-term environmental sustainability.

The European Union’s (EU) green transition has been driven by a series of landmark legislations and agreements, beginning with the Renewable Energy Directive (RED) ( European Parliament and Council, 2023a), and followed by RED II, which further strengthened the promotion of renewable energy in line with updated sustainability targets ( European Parliament and Council, 2018). Most recently, Directive (EU) 2023/2413 updated RED II by setting more ambitious renewable energy targets and accelerating the energy transition ( European Parliament and Council, 2023a). In parallel, the EU’s long-term decarbonisation strategy was outlined in the Energy Roadmap 2050, published in 2011, which aimed to reduce greenhouse gas emissions by 80–95% by 2050 compared to 1990 levels ( European Commission, 2011). This vision was later integrated and strengthened under the European Green Deal and its Adaptation Strategy ( European Commission, 2019). The RED framework and its subsequent updates provide the legal foundation for the energy transition by removing barriers to renewable deployment, promoting renewable heating, cooling, and transport fuels, and supporting investment in cost-effective technologies ( Plan Bleu, 2022). The 2030 Energy and Climate Framework, established in 2014, set targets to reduce emissions by at least 40%, increase renewable energy use, and improve energy efficiency by 27% by 2030 ( European Commission, 2014). These targets have been strengthened under the Fit for 55 package to align with the EU's goal of reducing net greenhouse gas emissions by 55% by 2030 ( European Commission, 2023e). A 90% reduction in transport-related emissions is also needed, alongside a targeted increase in inland waterways and short-sea shipping by 25% by 2030 and 50% by 2050, though these measures may impact marine and coastal environments ( European Commission, 2019). More recently, the Clean Industrial Deal ( European Commission, 2025a) outlined a roadmap to boost industrial competitiveness and accelerate decarbonisation through renewables, electrification, and circular economy practices by 2030, while COP29 reaffirmed global commitments under the Paris Agreement to accelerate the energy transition, phase out fossil fuels, and limit global warming to well below 2 °C, striving for 1.5 °C ( UNFCCC, 2025). These policy initiatives and legislative frameworks create the context in which renewable energy technologies, particularly wind energy, are increasingly central to the EU’s strategy for achieving climate and energy targets.

Wind energy currently represents the EU’s second-largest source of power generation capacity, following gas installations ( deCastro *et al.*, 2019). Within the wind energy sector, a key distinction exists between onshore and offshore systems. Onshore wind farms, located on land, have historically dominated Europe’s wind energy development, benefiting from relatively lower installation costs and established infrastructure. However, their further expansion increasingly faces challenges, including limited available land, social acceptance issues, and variable wind conditions, which can constrain generation efficiency and overall output ( Bilgili *et al.*, 2011). By contrast, offshore wind, implemented through offshore wind farms (OWFs), is becoming a key pillar of the EU’s strategy to achieve its energy and climate goals. Offshore turbines are installed at sea using foundation systems specifically designed to suit varying water depths and seabed conditions. Fixed-bottom foundations are suitable for shallow waters, typically up to 50 meters deep, while floating platforms are necessary in deeper areas ( Nikitas *et al.*, 2019). Since around 80% of global offshore wind resources lie in waters deeper than 50 meters, floating turbines are essential for unlocking the majority of this potential ( WindEurope, 2023), and can also reduce visual impacts and limit interference with maritime and fishing activities ( Farr *et al.*, 2021; Le Visage, 2022). Compared with onshore installations, offshore systems benefit from stronger and more consistent wind resources, which enable higher capacity factors and more stable energy generation ( Bilgili *et al.*, 2011).

Recognising this potential, the European Commission launched a strategy in 2020 setting a target to expand offshore wind capacity from 12 GW to 60 GW by 2030 and to 300 GW by 2050 ( European Commission, 2020a). A revised strategy in October 2023 raised the 2030 target to 111 GW and introduced measures to streamline permitting, enhance regional cooperation, and strengthen supply chains ( European Commission, 2023c). Beyond contributing to climate goals, the expansion of offshore wind can also reduce fossil fuel imports, improve air quality, and help mitigate climate-related risks ( WindEurope, 2023) The Mediterranean region presents significant potential for offshore wind development, particularly through floating technology. Although no commercial OWFs are currently operational in the area ( Plan Bleu, 2022), interest is growing due to favourable wind conditions, declining technology costs, and increasing international attention. To date, only a few pilot projects have been implemented, marking the early stages of offshore wind development in the region ( Lloret *et al.*, 2022). The predominance of deep waters makes fixed-bottom foundations largely unfeasible, positioning floating wind as the most suitable technological solution ( Martinez & Iglesias, 2021). Floating offshore wind could meet nearly 30% of the Mediterranean’s total energy demand, with Libya, Tunisia, Italy, and Greece together accounting for over 70% of this technical potential ( Faraggiana *et al.*, 2024). Many of these sites already demonstrate competitive energy costs, reinforcing the viability of floating wind as a key component of the Mediterranean’s renewable energy mix. However, current development remains limited, partly due to challenges such as social acceptance, maritime spatial planning, grid connectivity, and supply chain readiness. These factors are currently shaping the pace and direction of offshore wind development in the Mediterranean, underscoring the importance of ongoing coordination among EU Member States to align projects with existing national and regional energy strategies.

In France, several floating wind farms are under development in the Golfe du Lion off the southern coast (Occitanie region), notably the “Éoliennes Flottantes du Golfe du Lion” (EFGL) project, which comprises pilot and pre-commercial sites along with two additional pilot projects ( Gouty, 2022). Similarly, Italy is actively advancing floating offshore wind projects, particularly around Sicily, Sardinia, and Apulia, with government-supported initiatives reflecting a strategic emphasis on floating technology ( Watson Farley & Williams, 2025). Spain’s offshore wind activities have recently shifted focus toward the Mediterranean, particularly in the Balearic and Catalan regions, where floating wind pilot projects are being developed to address the challenges posed by deeper waters. Notably, Spain is advancing the “Parc Tramuntana” Floating Offshore Wind Farm Project in this area ( Diez-Caballero *et al.*, 2022). Greece has also recently enacted legislation to facilitate offshore wind development, with maritime spatial planning underway to identify suitable zones predominantly for floating wind farms in the Aegean, Ionian, and certain Mediterranean-facing coastal areas ( Alma Economics, 2021). These initiatives align with national plans outlined in the 2023 Maritime Spatial Planning Directive (MSPD) amendment ( European Parliament and Council, 2023b).

However, due to the Mediterranean Sea’s unique characteristics, assessments of potential offshore wind impacts remain largely speculative in the absence of operational examples. Most research to date has focused on OWFs in the North Sea, Baltic Sea, and North Atlantic, regions that differ significantly from the Mediterranean in terms of shelf morphology and the presence of sensitive benthic habitats, such as seagrass meadows and coralligenous reef ( Lloret *et al.*, 2022). In particular, the impacts of deep-water floating infrastructure, which is now central to Mediterranean deployment plans, are insufficiently understood. The limited and fragmented research available for the Mediterranean has fostered an overly optimistic perception of offshore wind’s contribution to the green transition, often overlooking region-specific environmental and socio-economic risks. Much of the current literature emphasises technical feasibility and economic potential, while long-term ecological and social implications remain underexplored ( Diez-Caballero *et al.*, 2022). Concerns include pollutant release from turbine materials, electromagnetic fields from subsea cabling, and underwater noise, alongside broader disruptions to marine ecosystems and coastal livelihoods ( Donázar-Aramendía *et al.*, 2025). While these issues have been studied in Northern European contexts, Mediterranean-specific conditions, such as its semi-enclosed geography, high biodiversity, and densely populated coastlines, necessitate a more tailored analysis.

This paper adopts a qualitative and reflective approach to examine offshore wind in the Mediterranean context. It first reviews the legislative framework governing offshore wind at the EU level, highlighting areas of limited applicability in the Mediterranean and identifying key regulatory and enforcement gaps. The analysis then considers the environmental, social, and economic challenges associated with offshore wind deployment in the region, drawing on recent studies, policy documents, and pilot projects. Synthesising these perspectives, the paper explores how context-specific governance and integrated policy approaches could shape the balance between decarbonisation objectives and environmental and social safeguards.

## Integrated marine governance in the Mediterranean

Since the 1992 Rio Declaration on Environment and Development ( United Nations, 1992), the international community has intensified efforts to promote the sustainable use of marine resources. The Declaration remains a foundational legal and policy instrument, having established core principles, such as the precautionary principle, polluter pays, and common but differentiated responsibilities, that continue to shape global and regional environmental governance frameworks today. A notable advancement in this regard was the adoption of Sustainable Development Goal 14 in 2015, which seeks to “conserve and sustainably use the oceans, seas, and marine resources for sustainable development” ( United Nations General Assembly, 2016). In parallel with these global commitments, the Mediterranean Action Plan (MAP) was instituted in 1975 as a multilateral environmental agreement under the auspices of the United Nations Environment Programme (UNEP). Its principal aim has been the protection of the marine environment through a harmonised regional framework. Over subsequent decades, UNEP/MAP has evolved into a model of global governance, instrumental in the negotiation and adoption of the Convention for the Protection of the Marine Environment and the Coastal Region of the Mediterranean (Barcelona Convention) and its related protocols. With seven protocols under the MAP, the Barcelona Convention is the region’s key legally binding multilateral environmental agreement ( UNEP/MAP, 1995), which came into force in 2004. Its Contracting Parties (CP) consist of 21 Mediterranean countries and the EU, encompassing the Mediterranean Sea eastward from the Strait of Gibraltar. The Convention allows each party to extend its application to its national territory, ensuring a broad and adaptable scope for regional cooperation.

Cooperation mechanisms, such as those under the Barcelona Convention, aim to coordinate the use of maritime resources, strengthen governance, and foster economic growth while promoting sustainable practices ( Plan Bleu, 2022). These efforts align with broader frameworks of renewable energy policies and marine ecosystem protections. According to Art.4, the CPs agree to take all necessary measures, individually or jointly, to prevent, reduce, and eliminate pollution of the Mediterranean Sea and to protect the marine environment to support sustainable development ( UNEP/MAP, 1995). A key component is the Dumping Protocol, entered into force in 1978, and amended in 1995 ( PNUE/PAM, 1976). Excluding Montenegro, all CPs to the Barcelona Convention are also parties to this protocol, which prohibits dumping activities, incineration at sea, and waste disposal, except for specific materials ( PNUE/PAM, 1976). In 2021, at the 22nd Meeting of the Contracting Parties in Turkey, a decision was made to amend the Annex to the Dumping Protocol. These amendments, binding in the EU under Article 29 of the Barcelona Convention, revised the permit criteria for sea disposal, specifying the properties of the materials to be dumped, as well as the characteristics of dumping sites and approved deposit methods ( European Commission, 2021b). The update demonstrates the CPs’ commitment to minimising pollution and promoting a sustainable management of the Mediterranean Sea.

In line with this commitment, regional efforts are guided by the Mediterranean Strategy for Sustainable Development (MSSD), adopted during COP19 of the Barcelona Convention in 2016. The MSSD provides a framework to align regional, sub-regional, and national policies with the 2030 Agenda for Sustainable Development ( Mancini, 2022) and complements the EU Marine Strategy Framework Directive (MSFD) 2016–2025, which emphasises ecosystem protection as a cornerstone of both economic and environmental policy ( European Commission, 2020b). It reinforces this approach by focusing on achieving Good Environmental Status (GES) in marine and coastal environments. The MSFD promotes ecosystem-based management, coordinating efforts across 23 coastal and five landlocked EU states, as well as with non-EU countries, to sustainably manage a marine area of 5.72 million km ^2^ ( European Commission, 2020b). In 2018, the CPs of the Barcelona Convention adopted the Ecosystem Approach (EcAp) and agreed on a roadmap for its implementation in the Mediterranean. The EcAp is a strategy for managing land, water, and living resources in a coordinated manner, supporting their conservation and equitable use. It serves as the guiding principle for policy development and implementation under the Barcelona Convention, supporting the achievement of GES in the Mediterranean marine and coastal environment through management decisions informed by quantitative assessment and monitoring ( SPA/RAC, 2015). At the Mediterranean level, the EcAp and the MSFD are implemented in synergy and coherence, and they are among the most ambitious international frameworks for marine environmental protection ( European Commission, 2025b). Additional measures include Art.9 of the Integrated Coastal Zone Management Protocol (ICZM) and the Union for the Mediterranean’s 2015 Ministerial Declaration, all of which encourage ecosystem preservation. EU policies, such as the Water Framework Directive (WFD) and its updates, further reinforce these efforts by aiming to achieve good ecological status for water bodies. Despite delays from funding and implementation challenges, the WFD created a governance framework for integrated water management, reducing chemical pollution and connecting human activities to environmental impacts ( European Parliament and Council, 2000).

This integrated approach ensures marine ecosystems remain functional and resilient for future generations. After launching the world’s first global framework to finance a sustainable Ocean Economy ( European Investment Bank, 2018), the EU introduced its new strategy on Biodiversity for 2030, focusing on “making nature healthy again” ( Bassetti, 2021). The strategy boosts the protection of marine ecosystems by expanding the protected areas and stressing the importance of the EcAp for managing human activities at sea. Sustainable innovation presents significant opportunities in green ports, shipping, aquaculture, water management, conservation, recycling, and maritime ecotourism, all key to the region's future. Meanwhile, the EU Commission highlighted the benefits of sustainable blue economy activities, advocating for an international Blue Economy Sustainability Framework ( European Commission, 2021c).

However, according to the results of the 2020 report on the first implementation cycles of the MSFD, the EU framework for protecting the marine environment needs to be strengthened ( European Commission, 2020a). Indeed, GES is achievable only if the pressures on the marine and coastal environment decrease and, therefore, if the uses of the marine and coastal environment that cause these pressures are adjusted. Surprisingly, the notion of GES appears only once in the text of the European Green Deal and only once in the Commission’s Communication on the Sustainable Blue Economy ( European Commission, 2021a). This may indicate a persistent lack of integration of marine protection into broader policies or a dominance of economic priorities over marine conservation, even under the guise of climate change adaptation.

Recognising the scale and diversity of its natural heritage, the EU has developed extensive legislation to protect and enhance Mediterranean biodiversity. As part of these efforts, it has also approved a taxonomy defining criteria for economic activities that contribute to climate adaptation and prioritise adaptation solutions ( European Commission, n.d.). Central to this is the Nature Directives, which establish Natura 2000, a network of protected areas comprising Special Areas of Conservation and Special Protection Areas under the Habitats Directive ( Council of the European Communities, 1992) and the Birds Directive ( European Parliament and Council, 2009). Natura 2000 aims to conserve rare, threatened, and endemic species, including all wild bird species, through protection, management, and monitoring measures. The network currently covers 18% of the EU’s land area and 6% of its marine territory; however, significant gaps remain in marine coverage, and only around half of the sites have management plans with defined conservation objectives ( European Commission, 2014). The “Fitness Check” evaluation confirmed the overall effectiveness of the directives but highlighted the need for more consistent and practical implementation. In response, subsequent legislative advancements have strengthened marine protection within the Natura 2000 framework. In 2023, Implementing Decision (EU) 2023/2806 established standardised site-specific data formats, enhancing transparency in reporting among Member States ( European Commission, 2023b). Additionally, the Nature Restoration Law, adopted in June 2024, introduced legally binding restoration targets aiming to restore at least 20% of the EU’s land and marine ecosystems by 2030, focusing on Natura 2000 sites, and up to 90% of degraded habitats by 2050 ( European Parliament, 2024). These initiatives are complemented by the ongoing revision of the MSFD to strengthen the assessment and achievement of GES ( European Commission, 2025b). This regulatory framework provides essential guidance for the sustainable development of maritime economic activities, including OWFs, which must balance energy expansion with the protection of sensitive marine ecosystems.

## Balancing Renewable Energy and Biodiversity in the Mediterranean Blue Economy

Within this framework, the Blue Economy, encompassing traditional maritime activities such as fishing, aquaculture, and coastal tourism, alongside emerging sectors like renewable energy and marine biotechnologies ( European Commission, 2021a), must balance development with conservation priorities. The Sustainable Blue Economy focuses on clean technologies, renewable energy, and circular material flows to secure long-term social and economic stability within planetary boundaries ( World Wide Fund, 2015). It seeks to balance the optimal use of maritime natural capital with human and social capital, advancing sustainable development, social equity, and reduced ecological risks. The EU's taxonomy for climate change mitigation, outlined in Regulation (EU) 2020/852, provides a framework for determining whether economic activities and investments qualify as environmentally sustainable, including provisions for the marine environment ( European Parliament and Council of the European Union, 2020). The regulation focuses on transitioning to renewable energy, reducing fossil fuel reliance, and promoting carbon-neutral energy sources. It allows activities with no viable low-carbon alternatives as long as they limit global temperature rise to 1.5°C above pre-industrial levels. Such activities must comply with strict GHG emissions standards and support the development of low-carbon alternatives. In June 2023, the Commission adopted delegated acts expanding the Taxonomy Regulation beyond climate objectives to include technical screening criteria for four additional environmental goals, including the sustainable use and protection of water and marine resources ( European Commission, 2023d).

Commission Delegated Regulation (EU) 2023/2485 establishes the general framework for determining whether an economic activity and its investments qualify as environmentally sustainable and includes several chapters related to the marine environment ( European Commission, 2023a). It provides technical criteria to determine when economic activities make a substantial contribution to climate change mitigation or adaptation while ensuring they do not harm other environmental objectives. It also emphasises the restoration of wetlands and the development of renewable energy sources, including wind, ocean, hydropower, geothermal, and cogeneration technologies. Furthermore, it states that offshore wind projects must not hinder achieving GES as defined by the MSFD, requiring measures to prevent or mitigate impacts on biodiversity and seabed integrity, supported by an environmental impact assessment and necessary mitigation and compensation measures. However, the references to the proper use of offshore wind turbines contained in the Regulations are insufficient to address the wide-ranging environmental impacts of these systems, including harm to marine ecosystems, pollutant emissions during their creation and dismantling, and the potential presence of environmentally hazardous substances in their buoys or mooring systems.

Natura 2000 sites, including Special Protection Areas for birds, are among the EU’s most biodiverse and valuable areas and must be protected from OWFs developments. Damage to these sites risks their legal status and may result in condemnation of member states ( Gouty, 2021). Under the Habitats Directive, wind farms within Natura 2000 sites are not prohibited; however, each project must undergo a case-by-case assessment to ensure it does not cause significant adverse effects, such as habitat loss, degradation, fragmentation, species disturbance, or other indirect impacts on environmental quality ( Fabrégat, 2021). Such assessments must consider the scale and magnitude of impacts and ongoing projects in the area. When appropriately integrated with other economic activities, including other renewable energy sources, the negative impacts of OWFs could be reduced, and biodiversity may even benefit. However, there is no current legal basis at the national or EU level to guarantee this outcome. The European Commission’s guidance document on wind energy developments in Natura 2000 sites aims to align with updated EU renewable energy and offshore wind policies ( European Commission, Directorate-General for Environment, 2020). It clarifies the application of Natura Directives and updates the 2011 guidance on wind farms in protected areas, emphasising the need for further knowledge, best practices, and policy integration. Nonetheless, as EC communications are non-legally binding ( EUR-Lex, 2007), this guidance serves primarily an informational role and lacks enforceability ( European Parliament and Council of the European Union, 2020). This limits its credibility and legal weight, underscoring the necessity for thorough case-by-case analyses prior to OWF development and revealing gaps in EU legislation where advances in renewable energy may conflict with biodiversity protection goals.

## Offshore wind: fixed vs floating environmental challenges

The EU legal framework supports the environmental sustainability of offshore renewable energy but remains broad and insufficiently tailored to the ecological pressures of offshore wind. High-level objectives, such as greenhouse gas reduction and biodiversity protection, do not fully account for differences between fixed-bottom and floating turbines, a gap that is especially critical in the Mediterranean, where complex ecosystems and competing maritime uses increase environmental and regulatory stakes. OWFs contribute significantly to climate change mitigation by reducing greenhouse gas emissions, yet they also carry ecological risks throughout their lifecycle, from raw material extraction and construction to operation, maintenance, and decommissioning ( Garcia-Teruel *et al.*, 2022).

The sector has evolved from fixed-bottom turbines in shallow waters up to 60 m deep to floating turbines in depths exceeding 120 m, with technological developments exploring installation in waters as deep as 1,000 m ( Farr *et al.*, 2021). Each configuration presents distinct ecological pressures: fixed turbines, sited closer to shore, pose higher risks of habitat loss, seabed disturbance, and species disturbance; whereas floating systems, deployed further offshore, may have broader spatial impacts through anchoring, mooring, and cable infrastructure, often in areas with limited ecological data and weaker regulatory oversight. Balancing offshore wind expansion with biodiversity conservation is therefore essential, particularly in areas overlapping sensitive habitats protected under Natura 2000 and other EU frameworks. Accelerating deployment requires aligning legal frameworks with technological realities through technology-specific impact assessments, adaptive management, and robust ecological monitoring to ensure renewable energy expansion does not compromise marine ecosystem protection. Table 1 provides an overview of the main environmental risks associated with each technology, highlighting the need to align regulatory safeguards with technological realities.

**Table 1.** Environmental Challenges of Offshore Wind Farms - Fixed vs. Floating. Summary of key environmental risks associated with fixed-bottom and floating offshore wind technologies. Source: Author's own synthesis based on existing literature.

Impact Category	Fixed Offshore Wind Farms (Shallow Water ≤60 m depth, anchored to seabed)	Floating Offshore Wind Farms (Deep Sea >60 m depth, moored floating platforms)	Cross-Cutting Concerns
**1. Seabed ** **& habitat** ** disturbance**	• Disturb ecologically important habitats such as Maerl beds and seagrass meadows	• Mooring and anchoring systems can disturb undisturbed deep-sea habitats.	• Habitat loss and fragmentation
**2. Biodiversity & ** **species impacts**	• Bird and bat collisions • Marine mammal disturbance • Reef and Reserve effects	• Increased mobility and deeper deployment of floating OWFs may intensify negative effects in vulnerable ecosystems.	• Wildlife interactions and ecosystem sensitivity
**3. Pollution**	• Chemical Pollution: leaching of heavy metals from sacrificial anodes and sediment disturbance • EMF & Thermal Effects from underwater cables can disorient marine species. • Accidental Risks: ship collisions, fires can cause oil or chemical spills.	• Greater presence of subsea cables increase cumulative EMF and thermal emissions, affecting benthic and pelagic zones. • Cables exclude fishing activities across wide marine areas	• Subsea infrastructure impacts on marine ecosystems and activities
**4. Lighting &** ** noise**	• Lights from OWF construction and operation disrupt natural behaviour of nocturnal species • Construction noise, especially from pile driving, creates acoustic pollution that harm marine mammals	• Floating platforms require towing to shore for maintenance, increasing marine traffic and operational disturbances • Risks of spills, emissions and ballast water discharges	• Disturbance from increased human activity at sea
**5. Oceano-** **graphic** ** processes**	• Alteration of current patterns, turbulence, bathymetry, and trigger upwelling or downwelling	• Environmental effects are harder to predict → need for precautionary governance	• Uncertain impacts on oceanographic processes

## Environmental impacts of fixed offshore wind farms

Fixed-bottom offshore wind farms are typically installed in shallow coastal waters, which are often rich in marine biodiversity. These areas are also near communities already affected by the decline of traditional industries like shipbuilding, fishing, and tourism, making them particularly sensitive to further change. Visual impacts on the seascape remain a major concern for residents and policymakers, with recreational users such as fishers, boaters, and beachgoers often expressing concern about changes to the coastal landscape. In the Mediterranean, large offshore wind proposals have faced strong opposition from local communities, tourism businesses, environmental NGOs, and regional authorities ( Lloret *et al.*, 2022).

### 1. Seabed & habitat disturbance

One of the primary environmental challenges is the disturbance and degradation of sensitive habitats such as Maerl beds and seagrass meadows, notably those formed by *Posidonia oceanica*, a protected and ecologically important species ( Barberá *et al.*, 2003). These ecosystems provide essential shelter, nursery areas, and carbon sequestration functions, and their disruption can lead to a cascade of ecological consequences ( Telesca *et al.*, 2015).

### 2. Biodiversity & species impacts

The risk of bird and bat collisions with turbine blades is a significant concern, especially for migratory species ( UNEP/MAP, 2022). Seabirds in the Mediterranean region, many of which are endangered, may have their migration routes altered or interrupted due to the placement of turbines ( Conseil national de la protection de la nature, 2021). In addition, nocturnal species such as bats are vulnerable to direct collision and displacement effects ( Richardson *et al.*, 2021).

Introducing artificial substrates through turbine foundations may lead to the formation of artificial reefs, generating what is known as the “reef effect” ( Danish Energy Agency, 2006). This phenomenon can benefit local biodiversity by attracting marine species in otherwise homogenous sandy habitats; however, it may also facilitate the spread of invasive species and lead to the exclusion of some native or mobile species ( Plan Bleu, 2022).

Fixed OWFs can act as de facto marine reserves due to restricted fishing activity in their vicinity. This “reserve effect” may enhance local fish stocks, but overfishing at the edges of these zones may reduce or negate broader conservation benefits, limiting the so-called “spillover effect” ( Lacroix & Pioch, 2011).

### 3. Pollution

Pollution from construction and operations, including heavy metal leaching from sacrificial anodes, threatens marine life, forming toxic compounds and degrading water quality ( Kirchgeorg *et al.*, 2018). Similarly, the remobilisation of sediments during geotechnical work can release trapped nutrients or pollutants ( Wang *et al.*, 2022), further impacting water column chemistry and benthic communities.

The electromagnetic fields (EMFs) and thermal emissions from underwater power cables used to transmit electricity to shore can disrupt species sensitive to electric and magnetic cues ( Emeana *et al.*, 2016). Fish, elasmobranchs, and other organisms that rely on geomagnetic navigation or electroreception may experience disorientation and altered behaviour ( Donázar-Aramendía *et al.*, 2025).

Accidental events such as ship collisions, fires, or extreme weather events can lead to chemical or oil spills, introducing acute pollution risks to marine ecosystems ( Arisian *et al.*, 2017). These events highlight the importance of risk management and emergency response planning at OWF sites.

### 4. Lighting & noise

Artificial lighting associated with OWFs construction, maintenance, and nighttime operation presents a visual disturbance that can disorient nocturnal marine species ( Farr *et al.*, 2021), such as seabirds and sea turtles. These lights attract animals to turbine sites, increasing the risk of collision and altering natural movement patterns ( Defingou *et al.*, 2019).

Underwater noise generated during construction, particularly from pile driving, represents a significant source of acoustic pollution ( Lindeboom *et al.*, 2015). Marine mammals, which rely heavily on sound for navigation, communication, and foraging, are especially susceptible to noise disturbance, which may result in physical injury or long-term behavioural changes ( Carrillo, 2010; Solangi, 2019).

### 5. Oceanographic processes

OWFs influence local oceanographic processes. Turbine structures can modify current patterns, induce upwelling or downwelling, and alter turbulence and bathymetry ( Lindeboom *et al.*, 2015). These changes can have cumulative ecological effects, mainly when multiple farms are clustered together.

## Environmental impacts of floating offshore wind farms

Floating offshore wind technology offers several environmental advantages compared to fixed system, including reduced visual impact due to deployment farther from shore and avoidance of shallow coastal habitats. Promising sites for floating offshore wind development in the Mediterranean have been identified primarily in the Gulf of Lions and the Aegean Sea, where favourable wind resources and comparatively low levelised costs of energy (around €95/MWh) contrast with higher costs in less optimal areas such as off the coast of Brindisi ( Faraggiana *et al.*, 2024). However, floating systems also present their own set of environmental concerns.

### 1. Seabed & habitat disturbance

Mooring and anchoring systems, such as suction piles or drag-embedded anchors, can disturb previously undisturbed deep-sea habitats. These habitats are often poorly understood and may harbor fragile ecosystems susceptible to mechanical disruption ( Garcia-Teruel *et al.*, 2022).

### 2. Biodiversity & species impacts

As with fixed systems, floating OWFs may generate artificial reef effects and facilitate the spread of non-native species. However, their increased mobility and deeper location could exacerbate these dynamics in less resilient ecosystems ( Farr *et al.*, 2021).

### 3. Pollution

The need for longer and more complex subsea cable systems introduces greater cumulative EMF and thermal emissions, which may affect wider swaths of benthic and pelagic environments. These cables also contribute to excluding fishing activities over large marine areas ( Le Visage, 2022).

### 4. Lighting & noise

The operational footprint and environmental impacts associated with floating wind turbines can be significant, particularly because floating platforms may need to be towed to shore for major maintenance ( Garcia-Teruel *et al.*, 2022). This increases the frequency and scale of marine operations and raises risks of spills, emissions, or ballast water discharges ( Mendecka & Lombardi, 2019).

### 5. Oceanographic processes

Floating wind farms are increasingly being planned for deployment in deep offshore regions that have, until now, experienced limited industrial activity ( Farr *et al.*, 2021). Consequently, the environmental impacts in these areas are more difficult to predict, and there is a greater need for precautionary governance.

## Socio-economic challenges

Research and monitoring of the socio-economic impacts of OWFs in the Mediterranean remain limited, as these considerations are often overshadowed by environmental and techno-economic concerns. These impacts include effects on employment, economic growth, housing, local services, community well-being, and potential conflicts with established sectors such as tourism and fisheries ( Negro *et al.*, 2020). While the offshore wind sector is moving beyond the demonstration stage, large-scale deployment faces significant constraints, including supply chain bottlenecks, insufficient port infrastructure, logistical challenges, and a shortage of specialised installation vessels. As turbines grow larger and are sited further offshore, these barriers become more pronounced ( Torres *et al.*, 2023). Construction is also highly weather-dependent, requiring uninterrupted operations and substantial storage facilities, while high vessel rental costs and port limitations add further difficulties ( Vis & Ursavas, 2016). Addressing component, infrastructure, and logistics challenges is key to supporting offshore wind growth. Table 2 summarises key challenges, including impacts on communities, ecosystems, supply chains, and maritime governance.

**Table 2.** Key Socio-Economic, Environmental, and Strategic Challenges of Offshore Wind Development. Summary of key socio-economic, environmental, and strategic challenges of offshore wind development. Source: Author’s own synthesis based on existing literature.

Main Challenge Area	Key Issues	Examples / Impacts
**1. Socio-economic impacts on** ** coastal communities**	• Employment effects • Economic growth & local development • Pressure on housing & services • Tourism & recreation	• Job creation during construction & maintenance • Port infrastructure improvements • Housing pressure in coastal areas • Potential drop in tourism revenues due to visual/landscape changes • OWFs enhance eco-tourism branding
**2. Conflicts with traditional** ** sectors**	• Exclusion of fishing activities • Increased operational costs for fishers • Navigation safety & space competition	• Reduced income for small-scale fisheries • Higher fuel & insurance costs • Hindered scientific fishery surveys • Collision risks & rerouted traffic
**3. Strategic material ** **dependency & supply chains**	• Heavy reliance on REEs • High supply risk • Recycling limitations • Price volatility & strategic vulnerability	• Dependence on neodymium, dysprosium, etc. • REE supply chain bottlenecks • Limited recycling infrastructure • Exposure to geopolitical shocks
**4. Environmental risks from ** **resource extraction**	• Increased REE demand → push for seabed mining • Sediment disturbance, noise, light pollution • Ecosystem degradation	• Deep-sea mining of polymetallic nodules • Reduced oxygen, turbidity increase • Harm to deep-sea species & slow recovery
**5. Geopolitical & infrastructure** ** constraints**	• Overlapping EEZ claims • Maritime disputes • Militarisation & security concerns • Infrastructure & logistical bottlenecks	• EEZ disputes under UNCLOS • Seabed militarisation & drone monitoring • Port capacity limits, installation vessel shortage, weather dependency

### 1. Socio-economic impacts on coastal communities

From an economic perspective, offshore wind farms affect employment, gross value added, and key coastal sectors such as fishing, shipping, and recreation. Construction and maintenance phases create job opportunities, often supported by training programs and local supply chain development ( Durning *et al.*, 2020). These projects can also drive improvements in port infrastructure, providing additional socio-economic benefits during the development stage ( Plan Bleu, 2022). Nevertheless, the social impacts can include increased pressure on housing and overall quality of life in coastal communities. In some cases, developers offer housing assistance or mitigation measures to address these challenges.

Coastal tourism may benefit from the presence of wind farms, which can be seen as symbols of clean energy. Site visits could be promoted to showcase collective planning and stakeholder engagement, while scientists might use the infrastructure for monitoring and research ( Lacroix & Pioch, 2011). At the same time, tourism, an essential economic driver in the Mediterranean, particularly for activities such as sailing, diving, swimming, and wildlife observation, may also be negatively affected. Offshore wind developments can restrict access to key areas and alter the visual seascape, potentially reducing tourism revenues ( Lloret *et al.*, 2022). Given the narrow continental shelves of many Mediterranean coastal states, OWFs are often situated relatively close to shore. This proximity may negatively affect the visual character of the seascape, deterring tourists and raising concerns about potential economic losses within affected communities ( Westerberg *et al.*, 2011). In response, Community Benefit Agreements have been introduced to recognise local involvement, offering benefits not as compensation but as a gesture of appreciation for supporting projects of national interest ( Durning *et al.*, 2020). Community acceptance remains crucial, as residents may also perceive OWFs as symbols of marine industrialisation, amplifying broader economic concerns.

### 2. Conflicts with traditional sectors

The exclusion of fishing from OWF zones poses a significant threat to small-scale fisheries, which dominate many Mediterranean coastal areas. Offshore wind developments generate multiple economic pressures on the sector, including higher fuel costs from displaced activities, reduced income and job security, increased insurance premiums due to navigational risks, and negative effects on related businesses ( Chaji & Werner, 2023). Installation and operational disturbances can further reduce fishery productivity, deepening existing economic vulnerabilities. These restrictions also hinder scientific fishery surveys, which are crucial for long-term ecosystem monitoring. At the same time, exclusion zones can function as de facto marine reserves, supporting biodiversity, though the effectiveness of buffer zones with MPAs remains poorly understood and warrants further research. Critically, the most technically suitable sites for OWF deployment often overlap with MPAs due to favourable wind and bathymetric conditions ( Lloret *et al.*, 2022), raising concerns that energy developers may prioritise technical feasibility over ecological sensitivity.

The Mediterranean’s status as one of the world’s most heavily trafficked seas further complicates OWF deployment. The installation of wind farms may reduce navigable space, increase collision risks for commercial and passenger vessels, and redirect traffic onto already congested routes ( Le Visage, 2022).

### 3. Strategic material dependency & supply chains

The generation of wind energy depends on materials associated with high supply risks. Modern turbines employ advanced permanent magnet (PM) generators and lightweight, fatigue-resistant blades designed for high efficiency and durability. Turbine components are typically constructed from steel alloys incorporating nickel, manganese, titanium, and other elements, while blades are composed of fibreglass and resin ( Hund *et al.*, 2020). Critically, the production of these technologies is heavily reliant on rare earth elements (REEs) such as neodymium and dysprosium, resources that are largely imported from outside the EU, with China and Latin America accounting for the majority of supply ( Bobba *et al.*, 2020). Demand for these materials is projected to increase substantially in the upcoming years.

Although the processing of raw materials remains environmentally intensive, emerging technologies offer opportunities to enhance both competitiveness and sustainability. For instance, iron nitride-based PMs, which utilise more abundant materials and offer cost advantages, are expected to become commercially viable in the near future ( Cui *et al.*, 2018). Similarly, advancements in recycling techniques for REEs could substantially reduce environmental impacts. While recycling REEs remains challenging at every stage, recovering them from consumer materials presents a promising alternative to conventional extraction methods ( Balaram, 2019). However, to capitalise on these developments, the EU must implement robust legislation to support material collection, recycling infrastructure, and transparent labelling systems for PM content ( Hund *et al.*, 2020). Despite progress, complex component separation and limited data on REE content in end-of-life products make recycling costly and inefficient. Continued innovation, policy support, and industry collaboration are essential for sustainable material cycles.

This dependency raises concerns about the EU’s strategic autonomy and the long-term viability of its energy transition, as the decreasing cost of renewable energy is offset by the significant environmental impacts associated with raw material extraction and processing. Climate-smart mining practices are essential to avoid shifting emissions from energy production to raw material extraction, thereby ensuring that the environmental benefits of wind energy are not undermined by unsustainable sourcing. Moreover, wind turbine costs are highly sensitive to fluctuations in metal prices, particularly REEs, due to rising global demand and the consolidation of smaller mining operations ( Cui *et al.*, 2018). The wind technology supply chain remains vulnerable at multiple stages, from raw material sourcing to component manufacturing. China’s near-monopoly on REEs and PMs is a strategic concern, as demonstrated by the 2011 market restrictions that triggered global price volatility ( Bobba *et al.*, 2020).

### 4. Environmental risks from resource extraction

In response, several European states have accelerated seabed exploration to identify domestic sources of REEs. Polymetallic nodules and cobalt-rich crusts in deep-sea environments are emerging as key sources of critical materials, drawing growing interest due to their vast metal content and mining potential ( Hund *et al.*, 2020). While seabed mapping aligns with UNESCO’s target of charting 80% of the ocean floor by the end of the decade, it often precedes commercial exploitation. Deep-sea mining raises serious environmental concerns, including sediment disturbance, noise, light pollution, and the introduction of invasive species ( United Nations Environment Programme, 2014). Exploring deep seabeds is often followed by exploiting these areas, which are still mysterious and widely unknown. However, deep-sea mining presents environmental threats. Extraction involves pulverising mineral crusts pumped to vessels, releasing pollutants, noise, light, and invasive species. The light affects deep-water species, while the altered water composition reduces oxygen and clouds the environment ( United Nations Environment Programme, 2014). These activities may significantly alter marine ecosystems, reduce oxygen levels, and impair water clarity. Deep-sea vent communities are especially vulnerable and can take years to recover from disturbance ( Joseph, 2017; Van Dover, 2011).

### 5. Geopolitical & infrastructure constraints

The strategic implications of OWF development are increasingly evident in the Mediterranean, where coastal states are asserting claims to marine resources through the establishment of Exclusive Economic Zones (EEZs), in accordance with the United Nations Convention on the Law of the Sea (UNCLOS) ( United Nations, 1982). Unlike hydrocarbon exploitation, offshore wind development relies on shared maritime jurisdictions ( deCastro *et al.*, 2019), a factor that in the Mediterranean’s complex geography has frequently led to overlapping EEZs and heightened disputes. Historically, Mediterranean states limited maritime claims to territorial seas and ecological protection zones. However, the discovery of fossil fuel deposits and the advancement of renewable energy have prompted a shift towards full EEZ declarations ( Pellen-Blin *et al.*, 2019). The ongoing tensions between Spain and France over such claims illustrate this broader challenge, even as both countries remain leaders in offshore wind development ( Le Visage, 2022). The seabed has become an increasingly contested space, marked by rising militarisation and security concerns for underwater infrastructure, exemplified by the French Navy’s deployment of deep-operating drones ( Kloetzli, 2022). Despite these tensions, joint management and cooperation would benefit regional stability and environmental protection.

## Discussion and conclusion

Climate change is a major driver of biodiversity loss in the Mediterranean, impacting both terrestrial and marine ecosystems. Although robust legal frameworks address land-based greenhouse gas emissions and environmental degradation, marine governance remains fragmented and poorly adapted to recent advances in marine renewable energy. This regulatory gap risks favoring climate mitigation over marine conservation, potentially compromising the achievement of GES and a genuinely sustainable Blue Economy in the region. OWFs are often promoted as key instruments of the green transition, yet their environmental and socio-economic impacts, particularly in the Mediterranean, remain underexplored ( Defingou *et al.*, 2019; Plan Bleu, 2022). The Mediterranean’s deep waters and ecological sensitivity make floating wind technology necessary, though it remains at an early stage. While wind energy is a cleaner alternative to fossil fuels, OWF construction and operation, both fixed and floating, pose risks such as underwater noise, habitat disturbance, pollution, and cumulative impacts, particularly in biodiversity-rich or poorly studied areas. Mitigation measures can reduce harm when combined with rigorous baselines, real-time monitoring, and long-term studies, especially as floating turbines are deployed at greater depths and scales.

Current legislative frameworks show inconsistencies and gaps that hinder effective risk assessment and mitigation. The lack of harmonisation across EU directives and limited coordination among Member States further exacerbate these challenges. To address them, comprehensive environmental impact assessments should be mandated at all project stages, from site selection to decommissioning, taking into account cumulative and synergistic effects. The application of the Precautionary Principle, embedded in the Treaty on the Functioning of the European Union and reflected in the MSFD and the MSPD, remains essential for safeguarding marine biodiversity ( European Parliament and Council, 2023b). Despite this, weak regulatory frameworks and insufficient research into socio-economic and environmental impacts continue to hinder the development of a sustainable offshore wind strategy in the Mediterranean. Protecting marine biodiversity and cultural heritage will require the expansion of marine protected areas and enhanced regional cooperation. Simultaneously, diversifying the EU’s REE supply chain is critical to reducing dependency on China, an effort that can be supported through strategic partnerships and exploration projects in countries such as Sweden, Finland, Germany, and Norway Bobba *et al.*, 2020).

Offshore wind energy offers significant potential to contribute to decarbonisation and sustainable development goals. Yet, without a more holistic, evidence-based, and precautionary approach, the sector risks replicating environmental harms it seeks to mitigate. The Mediterranean’s energy future depends on technological innovation, robust governance, environmental integrity, and collaborative regional management. As floating offshore wind expands into deeper Mediterranean waters, rigorous ecological baselines, long-term monitoring, and life-cycle studies of turbines are essential to ensure sustainability. Policy frameworks must balance energy transition goals with environmental protection, particularly in biodiversity-rich or poorly studied areas. International guidelines, pilot projects, and coordinated regional governance are needed to mitigate ecological and socio-economic risks. Managed effectively, offshore wind can contribute meaningfully to sustainable development goals. Otherwise, it may ultimately fall short of expectations as the “New Eldorado”.

## Ethical approval and consent

Ethical approval and consent were not required.

## Data Availability

No data are associated with this article. The table is based on the author’s synthesis of peer-reviewed literature and does not rely on a primary dataset. No external data are associated with the tables.
