# Supplementary material for: The Mediterranean Offshore Wind Turn: Lessons, Risks, and Regional Reflections
Source: Open Res Eur. 2025 Oct 22;5:66. Originally published 2025 Mar 7. [Version 3] doi: 10.12688/openreseurope.19339.3 (PMC12639279; doi:10.12688/openreseurope.19339.3)
Supplement: Peer review history

# Reviewer report: Reviewer response for version 3

**DOI:** 10.21956/openreseurope.23424.r62633

**Published:** 2025-11-21

**Authors:** Laura Castro-Santos

**Affiliations:** 1 Universidade da Coruna, A Coruña, Galicia, Spain

The paper is ready for indexing. Congratulations! Good job!

Is the argument persuasive and supported by appropriate evidence?

Partly

Is the work clearly and cogently presented?

Partly

Is the topic of the essay discussed accurately in the context of the current literature?

Partly

Does the essay contribute to the cultural, historical, social understanding of the field?

Partly

Reviewer Expertise:

NA

I confirm that I have read this submission and believe that I have an appropriate level of expertise to confirm that it is of an acceptable scientific standard.

# Reviewer report: Reviewer response for version 2

**DOI:** 10.21956/openreseurope.22521.r58114

**Published:** 2025-09-27

**Authors:** Daniel Mulas Hernando

**Affiliations:** 1 National Renewable Energy Laboratory, Colorado, USA

Summary:

 The manuscript examines the environmental, social, and economic implications of offshore wind energy (OWF) in the Mediterranean Sea, with particular attention to fixed-bottom and floating offshore wind technologies. It highlights ecological impacts, socio-economic considerations, material supply risks, and legislative and governance challenges, providing a qualitative assessment of the sector. The manuscript contains a substantial amount of relevant information and demonstrates awareness of current offshore wind technologies, policy frameworks, and Mediterranean regional context.

 Constructive Feedback:

1. Citation Gaps:
  - Several significant claims are presented without supporting references. For example, statements about biodiversity risks, EMF and thermal effects, artificial lighting impacts, reef/reserve effects, fisheries disruption, tourism impacts, and strategic dependencies on rare earth elements lack immediate citations.
  - Legislative and governance assertions regarding Mediterranean EEZ disputes and maritime militarisation require region-specific references.
2. Structure and Clarity:
  - The manuscript occasionally repeats information across sections, particularly regarding turbine technologies and environmental impacts. Structuring the “Environmental Challenges” section with enumerated points and separating fixed-bottom versus floating OWFs improves clarity, as suggested by other reviewers.
  - Flow between sections could be enhanced through clearer transitions or schematic diagrams illustrating interactions between environmental, socio-economic, and technical factors.
3. Technical Accuracy:
  - Some statements could mislead readers regarding commercially available technologies. For instance, two-bladed floating turbines and extremely large (>15 MW) turbines exist primarily at the prototype stage. Clarifying commercial readiness would strengthen the manuscript’s credibility.
4. Regional Context:
  - While the manuscript references the Mediterranean broadly, more precise information on suitable locations for fixed and floating OWFs and their environmental sensitivity would increase relevance.
  - Socio-economic impacts, including tourism and fisheries, should be supported by region-specific evidence to distinguish Mediterranean conditions from global generalizations.
5. Recommendations for Improvement:
  - Provide references for all significant claims, especially those highlighted above.
  - Use enumerated lists and sub-sections to clearly differentiate environmental impacts of fixed vs. floating offshore wind.
  - Clarify which turbine technologies are commercially deployed versus experimental or prototype.
  - Include region-specific socio-economic and ecological evidence where possible.
  - Consider including flowcharts or schematic diagrams to illustrate interactions and cumulative impacts.

Points That Must Be Addressed to Make the Article Scientifically Sound:

- Ensure all claims are supported by appropriate, current references.
- Distinguish clearly between commercial and prototype technologies.
- Enhance regional specificity to reflect Mediterranean conditions accurately.

Is the argument persuasive and supported by appropriate evidence?

Partly

Is the work clearly and cogently presented?

Partly

Is the topic of the essay discussed accurately in the context of the current literature?

Partly

Does the essay contribute to the cultural, historical, social understanding of the field?

Partly

Reviewer Expertise:

wind energy techno-economic and financial analysis

I confirm that I have read this submission and believe that I have an appropriate level of expertise to confirm that it is of an acceptable scientific standard, however I have significant reservations, as outlined above.

# Response

**Published:** 2025-10-15

**Authors:** Marta Avesani

**Affiliations:** University of Groningen Department of Communication and Information Sciences, Groningen, Groningen, The Netherlands

Thank you for the feedback. The comments have been addressed as detailed below: 

 1) All statements regarding biodiversity, EMF and thermal effects, artificial lighting, habitat and fisheries impacts, tourism, and rare earth dependencies have been reviewed and are now supported by up-to-date, peer-reviewed or authoritative sources. Legislative and governance assertions concerning Mediterranean EEZ disputes and maritime militarisation have been substantiated with region-specific references, clarifying the legal and policy context.

 2) Repetitive information on turbine technologies and environmental impacts has been streamlined, and the "Environmental Challenges" section has been restructured using enumerated points, with fixed-bottom and floating OWFs clearly distinguished, following reviewer suggestions. A table summarising key socio-economic, environmental, and strategic challenges of offshore wind development has been added, with the discussion elaborating interactions among these factors. Flow between sections has been improved through clearer transitional lines.

 3) Statements on turbine technologies have been revised to reflect their level of commercial readiness accurately.

 4) Information on suitable locations for fixed and floating OWFs in the Mediterranean has been refined, with greater emphasis on environmental sensitivity. Socio-economic impacts, including effects on tourism and fisheries, are now supported by region-specific evidence.

# Reviewer report: Reviewer response for version 1

**DOI:** 10.21956/openreseurope.20929.r53384

**Published:** 2025-04-25

**Authors:** Laura Castro-Santos

**Affiliations:** 1 Universidade da Coruna, A Coruña, Galicia, Spain

The aim of the paper is to analyze the need of legislation in order to address environmental, social and economic aspects of offshore wind energy in the Mediterranean Sea.

 I have some comments:

 1. Page 6. " *When discussing wind energy, a key distinction is between* *energy generated by onshore and offshore wind farms. OWFs* *consist of multiple wind turbines that convert the wind’s kinetic* *energy into electricity". *The definition of OWF could be the same as Onshore wind farms. You need to explain that in offshore the energy is generated offshore.

 2. Page 7. " *Floating wind turbines, typically* *with horizontal axes and two or three blades, are anchored to* *the seabed by cables and towed into position." *They are anchored to the seabed using mooring (chain or fiber).

 3. " *Offshore wind turbines, typically installed at depths of up to* *50 meters,..." *Before you talked about floating offshore and now you are talking about offshore wind in general (up to 60 meters only fixed systems can be installed). You need to separate the analysis of fixed offshore wind and floating offshore wind, because floating has less impact on environment.

 4. In section "Environmental challenges" I think that you need to enumerate (with numbers) each of them, because you repeat a lot of issues during this section (such as the sacrificial anodes, etc.). I think that you need to separate between fixed and floating offshore wind.

 5. The title "Cost-beneficiaries analysis" is not clear, because you do not calculate it with equations. Therefore, the title should be changed.

 6. I think that the "Mediterranean" in the title is not clear because the text is very generic and it is not applied directly to Mediterranean Sea. Firstly, it is important to know the areas of the Mediterranean Sea where fixed and floating offshore wind can be developed and secondly, you need to explain the differences between this two types of offshore wind in order to introduce the environmental and social issues.

 Please, ensure that the text is reviewed considering all these comments.

Is the argument persuasive and supported by appropriate evidence?

Partly

Is the work clearly and cogently presented?

Partly

Is the topic of the essay discussed accurately in the context of the current literature?

Partly

Does the essay contribute to the cultural, historical, social understanding of the field?

Partly

Reviewer Expertise:

Offshore wind

I confirm that I have read this submission and believe that I have an appropriate level of expertise to confirm that it is of an acceptable scientific standard, however I have significant reservations, as outlined above.

# Response

**Published:** 2025-06-28

**Authors:** Marta Avesani

**Affiliations:** University of Groningen Department of Communication and Information Sciences, Groningen, Groningen, The Netherlands

I thank the reviewer for the constructive feedback. In response to the reviewer’s input regarding the definition of offshore wind farms (OWFs), the text has been revised to clarify that OWFs are located at sea and utilise marine wind resources to generate electricity. This clarification distinctly differentiates OWFs from onshore wind farms, which are situated on land.

 The description of floating wind turbines has been updated to accurately state that they are anchored to the seabed using mooring systems, which typically consist of chains or synthetic fibers, rather than cables.

 This revision ensures technical accuracy in describing the anchoring methods used for floating offshore wind technology.

 The text has been revised to clearly differentiate between fixed-bottom and floating offshore wind technologies, specifying their respective depth ranges and associated environmental impacts. This distinction provides a clearer and more accurate analysis by addressing the unique characteristics and effects of each technology type.

 The "Environmental challenges" section has been revised to clearly enumerate each challenge, improving the overall structure and reducing repetition. Additionally, the discussion has been separated to address the environmental impacts of fixed-bottom and floating offshore wind systems individually, ensuring a more precise and organized analysis. The title has been revised to reflect the qualitative nature of the analysis better, has been clarified to more accurately reflect the focus on the Mediterranean Sea, replacing the previous “Cost-beneficiaries analysis” with

**“The Mediterranean Offshore Wind Turn: Lessons, Risks, and Regional Reflections.”** The text has been revised to include a detailed description of specific areas within the Mediterranean that are suitable for both fixed and floating offshore wind development. Additionally, the technical differences between these two types of offshore wind technologies are explained to establish a clear foundation for the subsequent discussion of their environmental and social impacts. This revision ensures the analysis is both regionally relevant and technically grounded.

# Reviewer report: Reviewer response for version 1

**DOI:** 10.21956/openreseurope.20929.r52200

**Published:** 2025-04-10

**Authors:** Barry Johnston

**Affiliations:** 1 Atlantic Technological University, Galway, County Donegal, Ireland; 2 Queen's University, Belfast, UK

The title of the manuscript is *‘Cost-benefit analysis of the offshore wind power transition in the Mediterranean: Towards a “New Eldorado”?’*, however the manuscript does not provide a quantitative study on this topic, instead is a collection of qualitative data and observations on primarily how offshore wind energy may impact, biodiversity, supply chains and economic development. The manuscript has a substantial volume of relevant information, most of which is well structured. Where appropriate, the manuscript uses examples from existing offshore wind locations. It draws comparisons, considering that the EU member states are considering offshore wind deployment in the Mediterranean, which is a valuable exercise.

 Several issues should be addressed before the manuscript is suitable for acceptance.

- Firstly, a general observation is that several statements may be correct but are not supported by references in the text.

For example, ‘OWFs reduce emissions but can harm aquatic ecosystems, with biodiversity protection often overlooked in offshore energy areas. ’ This is a significant statement without any reference or supporting evidence.

 There are multiple other examples of this. Please review the manuscript thoroughly and ensure that data or a source supports any statement.

- The manuscript's structure does not always seem coherent. It would benefit from illustrations or flowcharts depicting how the author sees some of the interactions.

For example, the paper's structure leaves some duplication, where references to turbine technology appear in multiple sections. It may be worthwhile to reconsider the headings and structure of the manuscript so that sections fit together, and the manuscript flows better.

- Some of the references are not the most current.

For example, ‘2030 Energy and Climate Framework in 2014, which set targets to reduce emissions by at least 40%, increase renewable energy use, and achieve a 27% improvement in energy efficiency by 2030’. I don’t think this is current, and these targets have been revised.

 There are other examples of dated references. Please check the sources and ensure that the data presented is current.

- Some technical details about offshore wind as a technology need to be improved; some statements are open to misinterpretation.

For example, some text refers to technologies still in prototype stages and may not reach commercial development. Still, the manuscript reads as if these technologies are at commercially readily available.

 For example, page 7 reads ‘These turbines can have capacities of 15 MW or more, with blade lengths exceeding 100m, and are installed in clusters connected by seabed cables’. This is misleading, while turbines larger than 15MW exist, the majority deployed are less than this. Additionally, text refers to 2-blade floating turbines; again, these exist as prototypes, but the author needs to check when/if they will be deployed commercially and clarify this in the text.

- Some of the text makes unclear connections.

For example, ‘The new wind turbines, where a generator converts mechanical energy into electrical energy, are programmed to achieve maximum efficiency and the lowest possible energy costs. Fluctuating wind energy makes variable speed generators, like permanent magnet (PM) generators, and blades with high strength-to-weight ratios and fatigue resistance, the most suitable options.’

 While each element is likely true when considered in isolation, it is unclear what the author tries to say by connecting these points.

- The author has noted potential supply chain constraints and mentions ports in the context of a redevelopment opportunity; the reality is that the lack of port infrastructure and installation vessel availability is likely to create deployment limitations and should be noted and explored as a risk.
- It is unclear if there is an issue with the referencing system or typesetting. Still, many of the references in the footnotes are not complete, and it is difficult to follow the reference from the text to the source, as the numbering system is not chronological.

The examples provided in this review are not an exhaustive list but serve to provide examples. Please ensure that when revising this manuscript, the text is thoroughly reviewed to ensure that the principles detailed here are applied consistently across the manuscript.

Is the argument persuasive and supported by appropriate evidence?

Partly

Is the work clearly and cogently presented?

Partly

Is the topic of the essay discussed accurately in the context of the current literature?

Partly

Does the essay contribute to the cultural, historical, social understanding of the field?

Partly

Reviewer Expertise:

offshore wind energy, economic assessment, engineering assessment, resource assessment, financial analysis, policy mapping

I confirm that I have read this submission and believe that I have an appropriate level of expertise to confirm that it is of an acceptable scientific standard, however I have significant reservations, as outlined above.

# Response

**Published:** 2025-06-28

**Authors:** Marta Avesani

**Affiliations:** University of Groningen Department of Communication and Information Sciences, Groningen, Groningen, The Netherlands

I would like to thank the reviewers for their constructive feedback. In response, the title has been revised to:

**"The Mediterranean Offshore Wind Turn: Lessons, Risks, and Regional Reflections"**

 to more accurately reflect the manuscript’s qualitative focus. The manuscript has undergone a thorough review to ensure that all significant claims, including the example highlighted, are now fully supported by appropriate references and evidence. Structural revisions have been made to improve coherence and logical flow between sections, while reducing redundancy, particularly in the discussion of turbine technology. Additionally, illustrative elements have been added where relevant to clarify key interactions and enhance reader understanding. Thank you for the helpful observation regarding the currency of the references.

 The relevant sources and data have been carefully reviewed to ensure alignment with the most recent targets and policy frameworks. Outdated references have been replaced with more current sources where appropriate. In instances where earlier references provide important historical context, they have been retained and supplemented with updated data to ensure both accuracy and contextual depth.

 The relevant sections have been revised to more clearly distinguish between commercially deployed systems and those that remain at the prototype or demonstration stage. Statements that previously implied widespread commercial availability have been adjusted to reflect the current state of technological development. Specific examples, such as turbine capacity and the status of two-bladed floating turbines, have been clarified to prevent potential misinterpretation.

 Revisions have been made to improve the clarity and logical flow of the discussion on turbine design and related technologies. The updated section now more clearly articulates the relationship between energy efficiency, variable-speed generators, and the use of advanced blade materials, ensuring that the connections between these elements are technically accurate and easy to follow.

 The discussion has been expanded to address the risks posed by limited port infrastructure and the availability of installation vessels.

 These factors are now identified as critical supply chain constraints with potential implications for deployment timelines and overall project feasibility. The referencing system was significantly disrupted during the typesetting phase. All citations have been carefully reinserted, and the reference list has been reviewed to ensure completeness and correct formatting.

 The referencing style has also been converted to an in-text citation format to facilitate easier source tracing. Additionally, new references have been incorporated where necessary to support the revised content and strengthen the overall evidence base.
